# Behavioral and Transcriptomic Fingerprints of an Enriched Environment in Horses (*Equus caballus*)

**DOI:** 10.1371/journal.pone.0114384

**Published:** 2014-12-10

**Authors:** Léa Lansade, Mathilde Valenchon, Aline Foury, Claire Neveux, Steve W. Cole, Sophie Layé, Bruno Cardinaud, Frédéric Lévy, Marie-Pierre Moisan

**Affiliations:** 1 INRA, UMR85 Physiologie de la Reproduction et des Comportements, Nouzilly, France; 2 CNRS, UMR7247 Physiologie de la Reproduction et des Comportements, Nouzilly, France; 3 Université François Rabelais de Tours, Tours, France; 4 IFCE, Nouzilly, France; 5 INRA, Nutrition et neurobiologie intégrée, UMR 1286, Bordeaux, France; 6 Université Bordeaux, Nutrition et neurobiologie intégrée, UMR 1286, Bordeaux, France; 7 Division of Hematology–Oncology, Department of Medicine, University of California Los Angeles, Los Angeles, United States of America; 8 INSERM, U1035, Biothérapies des Maladies Génétiques et Cancers, Bordeaux and Institut Polytechnique de Bordeaux, Talence, France; University of Florida, United States of America

## Abstract

The use of environmental enrichment (EE) has grown in popularity over decades, particularly because EE is known to promote cognitive functions and well-being. Nonetheless, little is known about how EE may affect personality and gene expression. To address this question in a domestic animal, 10-month-old horses were maintained in a controlled environment or EE for 12 weeks. The control horses (n = 9) lived in individual stalls on wood shaving bedding. They were turned out to individual paddocks three times a week and were fed three times a day with pellets or hay. EE-treated horses (n = 10) were housed in large individual stalls on straw bedding 7 hours per day and spent the remainder of the time together at pasture. They were fed three times a day with flavored pellets, hay, or fruits and were exposed daily to various objects, odors, and music. The EE modified three dimensions of personality: fearfulness, reactivity to humans, and sensory sensitivity. Some of these changes persisted >3 months after treatment. These changes are suggestive of a more positive perception of the environment and a higher level of curiosity in EE-treated horses, explaining partly why these horses showed better learning performance in a Go/No-Go task. Reduced expression of stress indicators indicated that the EE also improved well-being. Finally, whole-blood transcriptomic analysis showed that in addition to an effect on the cortisol level, the EE induced the expression of genes involved in cell growth and proliferation, while the control treatment activated genes related to apoptosis. Changes in both behavior and gene expression may constitute a psychobiological signature of the effects of enrichment and result in improved well-being. This study illustrates how the environment interacts with genetic information in shaping the individual at both the behavioral and molecular levels.

## Introduction

Environmental enrichment (EE) is classically defined as a combination of complex inanimate and social stimuli. It involves provision of various sensory stimuli, novel objects to explore, social contacts, and a possibility of voluntary exercise. The effects of EE have been widely studied and are diverse: a reduction in anxiety [1], learning and memory enhancement [2]–[4], protection from neurodegenerative damage [5], facilitation of human–animal interactions, and improvement in the well-being of captive animals [6]–[8].

In spite of the numerous reported effects, very few studies have been conducted to assess the impact of EE on personality. The concept of personality (or temperament) refers to a set of individual differences in behavioral tendencies, called traits or dimensions, that are relatively stable across various types of situations and throughout the lifespan of the animal [9]. Personality is also shaped throughout the lifespan under the influence of environmental factors. Not all events can change personality, but given the numerous effects of EE previously described in the literature, we suspected that EE could affect some personality dimensions. An effect on dimensions related to anxiety or stress response was expected here [1], [4], but we also suspected larger behavioral changes, particularly in dimensions such as sensory sensitivity, activity, or gregariousness. The advantage of the horse for tests of the impact of EE on personality is that this concept has been extensively studied in this species [10], and five main dimensions can be characterized using behavioral tests: fearfulness, gregariousness, sensory sensitivity, activity level, and reactivity to humans [11]–[14]. Knowledge of these effects should help to determine how the environment interacts with genetic information in shaping the development of personality. In addition, given the links existing between personality and cognition [15], [16], we hypothesized that EE could also influence learning abilities, particularly in a task requiring focused attention.

A growing body of literature in recent years has shown that social–environmental factors experienced during the lifespan appear to exert a strong influence on the most basic internal biological processes, e.g., the expression of genes [17]. For example, human social genomic studies have revealed that several types of social adversity (e.g., social isolation, imminent bereavement, and low socioeconomic status) are linked to an increase in the expression of proinflammatory genes and downregulation of genes related to an antiviral immune response in circulating leukocytes [18]. Other pathways have been implicated in animal social signal transduction such as early growth response (EGR) signaling [19]. Because these changes are long-lasting, they can be used as a biological signature of the effects of negative or positive environmental conditions and may help to predict behavioral problems or a disease risk.

From the standpoint of ethics, a better understanding of the impact of housing conditions on behavior and gene expression is relevant to all animals living in captivity, whether they are laboratory, zoo, or farm animals, or pets. Horses are particularly relevant to this issue because they are often housed in an individual stall, deprived of voluntary physical exercise, or social and sensory stimuli. This is especially the case for performance horses such as race horses, show jumping horses, or those kept on small acreages (e.g., in urban riding centers). These housing conditions compromise their well-being [20], and render the horses highly reactive and thus insecure in relation to humans [21]. Although the effects of EE have been studied in horses previously [20]–[26], only one enrichment item was taken into account at a time in these studies, precluding assessment of the impact of combined inanimate and social stimuli.

The aims of the current experiment were to determine if a program of EE, including various components such as physical activity, sensory stimuli, and social contacts, would durably change personality of horses, enhance their learning abilities, and improve their well-being. In addition, we hypothesized that the expression of stress-related genes would be sensitive to EE and these changes could constitute a genomic signature of well-being. These aims were achieved, and overall, this study allowed us to determine a clear behavioral and transcriptomic signature of EE in horses.

## Methods

### Ethics Statement

All animal care procedures were in accordance with the guidelines set by the European Communities Council Directive (86/609/EEC) and with French legislation on animal research. The experiment was conducted under a license from the French Ministry of Agriculture (No. 37–125). The procedure reported in this paper was approved by the ethics committee of Val de Loire, and the horses belonged to the experimental unit (UEPAO) of the INRA of Nouzilly (permit No. delivered by the local ethics committee “CEEA VdL, Comité d'Ethique pour l'Expérimentation Animale du Val de Loire”: E 37-175-2). A minimal number of animals per group was used for statistical testing of differences. The housing conditions of the control animals corresponded to the normal housing conditions of the horses living indoors. At the end of the experiment, all the animals were returned to their normal housing arrangements, at pasture. From the standpoint of ethics, this experiment provided scientific data to promote the use of EE in the horse industry as well as on experimental farms, for improvement of well-being of horses.

### Animals and experimental groups

We analyzed 19 prepubertal Welsh foals (age: 10±1 months; average height: 1.08 m). They were weaned at 7 months of age, and were reared together until the start of the experiment. The horses were then randomly allocated to either the EE-treated group (*n* = 10, four males and six females) or the control group (*n* = 9, six males and three females), randomized by personality (see File S1 for details).

### Treatments

The treatment began at the start of week 1 and lasted 12 weeks. The amount of time that humans were present and handled the horses was strictly equal controlled in the two groups (EE and control). Water was available *ad libitum*. Feed rations were calculated to provide an equal energy value to both groups.

#### Control treatment

Horses were continuously housed in individual stalls (1.6 m×3.5 m) on wood shaving bedding. Three times a week, they were led by an experimenter with a halter to an individual dirt paddock (15 m×30 m) for 1 h. They could see, smell, and hear the other horses, but no physical contact was possible between them. They received 1 kg of concentrated pellets twice a day (morning and evening) and 2 kg of hay midday in a hay net.

#### EE treatment

Each day, from 0900 h to 1600 h, the horses were housed randomly in large individual stalls (4 m×5 m) on straw bedding. The rest of the time, they lived together at pasture, with an adult mare. They were led to the pasture or to the stall without being haltered (the doors were simply freed). In the morning and evening, the horses were fed with a meal randomly composed of 500 g of oat bran, 80 g of carrots, 70 g of apples, 50 g of alfalfa horse treats, or 500 g of concentrated pellets, randomly flavored each time with different feed additives: garlic, fenugreek, cumin, banana, cherry, or oregano. These pellets were placed randomly in a feed bucket (but hidden under straw), were provided in a chest covered with a nose-removable lid, or were scattered on the ground in the straw. At midday, they received 2.1 kg of two types of hay distributed in equal shares of 700 g in three hay nets of various colors attached to three sites within the stall.

During the entire experiment, a soft plastic brush, a hard sisal brush, a green hard rug (made of plastic bits), and a coconut rug were placed along the wall, at a height that allowed the horses to scratch their head, shoulders, and croup (1 m above the ground). Each week, four new and unfamiliar objects (e.g., a balloon, tire) were introduced into the stalls and two such objects at pasture. These objects were either placed on the floor or hung up 1.3 m above the ground. Five days a week, classical or country music was alternately broadcasted in the stable for 1 h. Each week, a new plastic bottle containing a compress soaked with essential oil of cinnamon, thyme, lavender, orange, or cloves was hung up in each stall. Finally, three times a week, the horses were led individually with a halter to different unknown locations for a period of 20 min (alternatively into two different stalls or to a small paddock where unfamiliar objects were placed).

### Behavioral observations in the home stall

**Figure 1:**
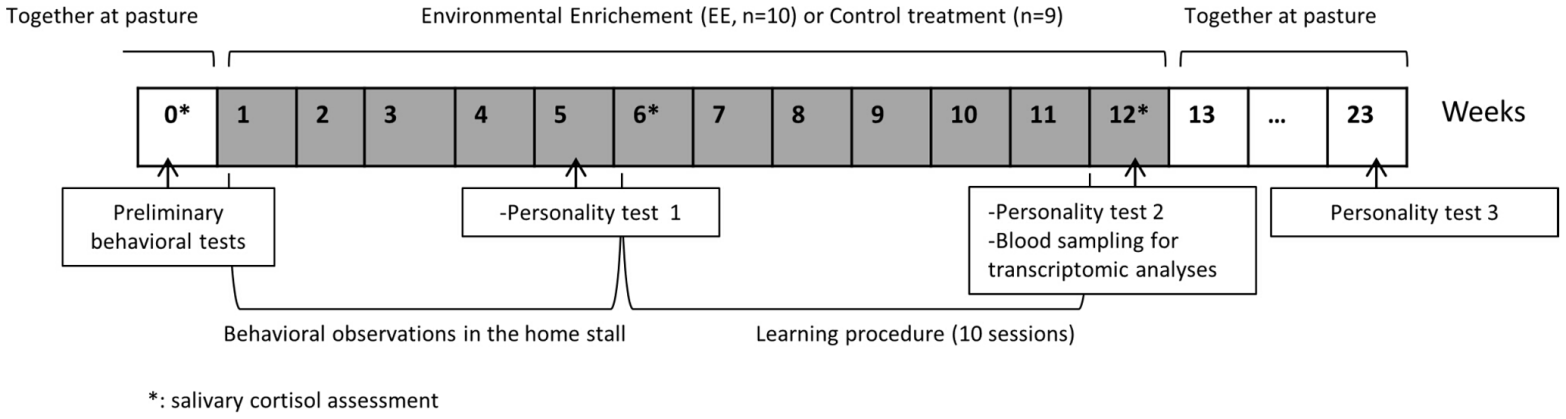
Schematic representation of the protocol. Each horse (10 months old) was subjected to either the environmental enrichment (EE; *n* = 10) or control treatment (*n* = 9) for 12 consecutive weeks.

During the first 5 weeks of the experimentation (Figure 1), a horse's behavioral patterns (see File S1) were examined using scan-sampling from Monday to Friday, 90 min/d.

### Personality tests

At the beginning of weeks 5, 12, and 23, each subject was led to an unfamiliar arena to be subjected to seven successive tests to assess five independent dimensions of personality: fearfulness, tactile sensitivity, gregariousness, the activity level, and reactivity to a human. At the beginning of week 5, an additional handling test was performed. The details are described in File S1.

### Learning

Ten learning sessions were conducted from the sixth to the eleventh week. The instrumental task consisted of a horse touching a traffic cone with its nose as instructed by an experimenter, in order to obtain a food reward. Two experimenters conducted the trials alternately. In the *shaping phase (A+, B+)* both experimenter A and B reinforced the animal's behavior with a food reward when it touched the suggested cone. In the *Go/No-go task (A+, B−)*, only one experimenter (A+), always the same, reinforced the animal's behavior, while the other (B−) never reinforced the behavior. The details are described in File S1.

### Cortisol measurements

Basal salivary cortisol concentrations were assessed at the beginning of week 0 (before treatment) and at the beginning of weeks 6 and 12. Each time, two samples were collected during a day without any testing procedure, except for the procedures related to the EE or control treatments: one morning sample was collected at 1000 h and one afternoon sample at 1530 h. The details are described in File S1.

### Sample collection for gene expression analysis

For transcriptomic analysis, 5 mL of venous blood was collected from 19 horses after 12 weeks of treatment, using vacuum tubes that contained a reagent that immediately stabilized intracellular RNA (PAXgene blood RNA system; PreAnalytiX GmbH, Hombrechtikon, Switzerland). The samples were maintained at room temperature for 8 h as required for stabilization of RNA and then at −20°C until RNA extraction.

### RNA extraction and microarray analysis

Total RNA was extracted using the PAXgene Blood RNA Kit (Qiagen, Courtaboeuf, France) according to the manufacturer's protocol. Quality of the total RNA was assessed and its concentration was measured using RNA Nano chips on a Bioanalyser 2100 (Agilent, Boeblingen, Germany). All the samples had an RNA Integrity Number (RIN) score of >8.0.

Cyanine-3 (Cy3)–labeled cRNA was prepared from 0.4 µg of RNA using the One-Color Microarray-Based Gene Expression Analysis Kit (Quick Amp Labeling, Agilent Technologies, Massy, France) according to the manufacturer's instructions, followed by RNeasy column purification (Qiagen, Courtaboeuf, France). Dye incorporation and the cRNA yield were verified using a NanoDrop 2000 spectrophotometer.

For each sample, 1.65 µg of cRNA was fragmented and hybridized overnight at 65°C onto the Horse-Genopole Microarray (custom Agilent 4×44K eArrays, AMADID 026033, Agilent Technologies, Massy, France) that included 43803 horse cDNA probes. The Horse-Genopole Microarray is the control Horse Gene Expression Microarray (AMAMID 021322, Agilent Technologies, Massy, France) enriched with 384 equine transcripts [27]. The slides were washed as recommended by the manufacturer and scanned on an Agilent G2565CA scanner, at 5-micron resolution in 20-bit scan mode (Agilent Technologies, Massy, France). The resulting images were processed using the Feature Extraction software (version 10.7). Raw data were deposited as Gene Expression Omnibus series GSE50623 on the website of the National Center for Biotechnology Information (NCBI).

### Statistical and bioinformatic analyses

#### Behavioral and physiological data

hapiro–Wilk tests of the collected data revealed a deviation from normality; thus, we used nonparametric statistics. Groups were compared using two-tailed Mann–Whitney tests. Intragroup comparison was conducted using Wilcoxon tests. The χ^2^ test was used to compare proportions between groups. All calculations were performed using the XLSTAT software (Addinsoft Software, Paris, France). The results are presented as a median and an interquartile range. The level of statistical significance was set to *P*<0.05.

#### Transcriptomic data

The data were normalized for interarray comparison and analyzed using the BRB-ArrayTools package (version 4.2.0, http://linus.nci.nih.gov/BRB-ArrayTools.html). As recommended by Agilent Technologies, we chose the percentile method for the normalization, and adjusted the 75^th^ percentile of all noncontrol probes to 500. We identified genes that were differentially expressed between the two groups using a random-variance *t* test (Class Comparison Between Groups of Arrays Package, BRB-ArrayTools). Gene expression differences were considered statistically significant if they were showing a≧20% difference in mean expression levels between samples from the EE and the control conditions, and if the resulting *P* value was <0.005.

Details of the bioinformatic analyses using TELiS, oPOSSUM, and Ingenuity Pathway Analysis are provided in File S1.

## Results

### Behavioral observations in the home stall

**Figure 2:**
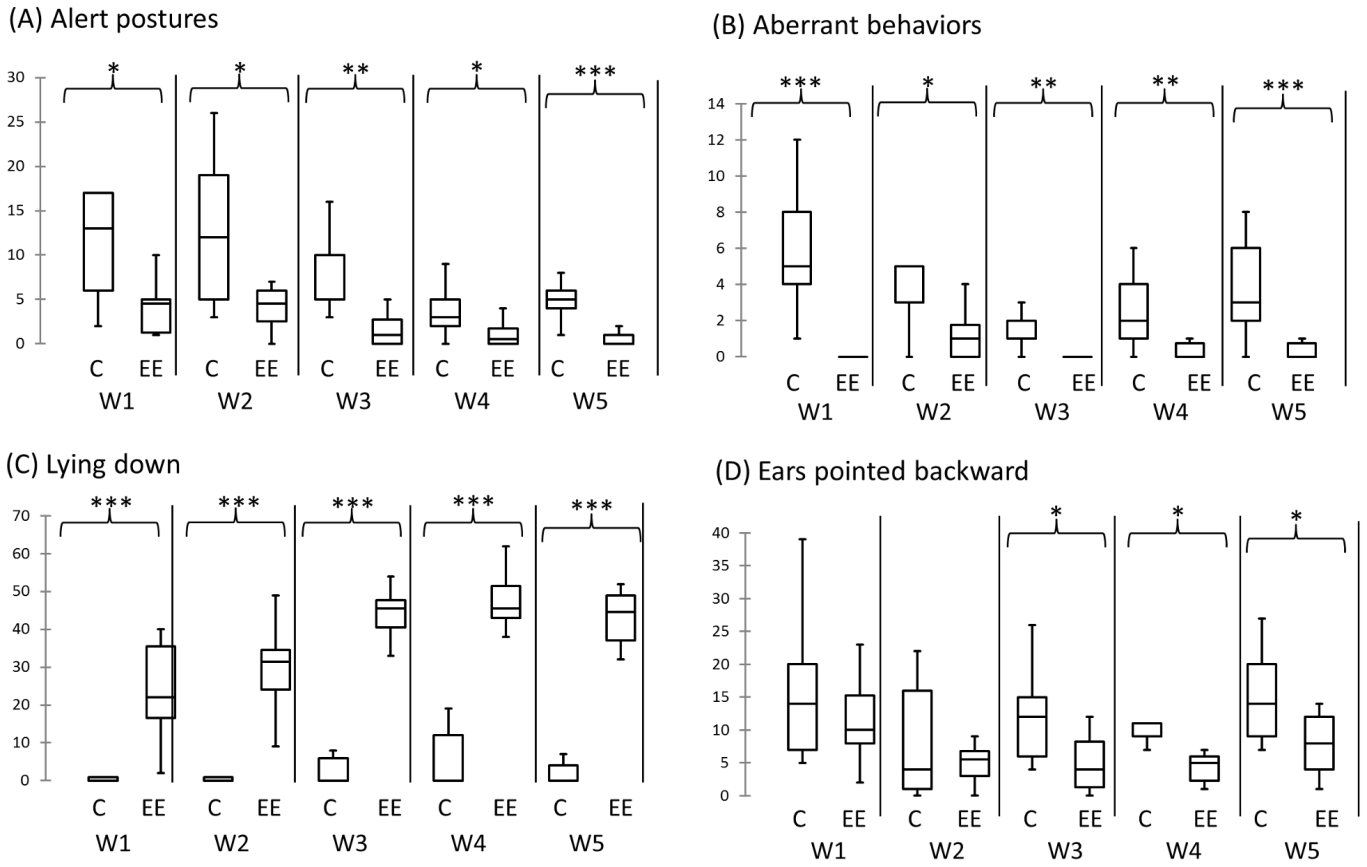
Behavioral effects observed in the home stall. Comparison between groups regarding the number of times the horses were observed in alert postures (A), with aberrant behavior (B), lying down, (C), and with the ears pointed backward (D) during scan sampling each week. C: Control horses, EE: environmentally enriched horses, W1–W5: weeks from the first to the fifth. *P<0.05, **P<0.01, and ***P<0.001 (Mann–Whitney test).

During the first week, EE-treated horses vocalized less frequently than did control horses (U = 22, *P* = 0.04). Each week, EE-treated horses exhibited alert postures and aberrant behavior less often and lied down more often than did horses maintained under control conditions (Figures 2A, B, and C). From the third to the fifth week, EE-treated horses displayed ears pointed backward less often than did the control horses (Figure 2D). During the first and the fifth week, all the horses finished their meals, in both groups. At the beginning of week 12, all EE-treated horses finished their meal, whereas four of the nine control horses did not (χ^2^ = 5.63; *P* = 0.018).

### Personality tests

The medians (interquartile) of the variables of personality assessed at the beginning of weeks 5, 12 and 23, and the *P* values are shown in Table 1. At week 5 (mid-treatment), EE-treated horses were significantly less fearful: they glanced at a novel object significantly less often than did the control horses and ate significantly more rapidly during the suddenness test. They also had lower tactile sensitivity judging by a significantly weaker reaction to stifle-haunch axis stimulation. They were significantly more often in contact with a passive human and were fitted with a halter significantly more rapidly. In the additional handling tests, they expressed fewer defensive reactions. At week 12 (immediately after the end of treatment), the EE-treated horses were still less fearful than control horses: the former were significantly more often in contact with a novel object, glanced at it significantly less often, and ate significantly more rapidly during the novel area test. They had a significantly lower rate of response to von Frey filaments, and a significantly weaker reaction to stifle-haunch axis stimulation. They were significantly more often in contact with a passive human and were fitted with a halter significantly more rapidly. At week 23 (3 months after the end of treatment), the EE-treated horses were again less fearful and less reactive to tactile stimuli than were the control horses: the EE-treated horses were significantly more often in contact with a novel object, glanced at it significantly less often, ate significantly more rapidly during the novel arena test, and had a significantly weaker reaction to stifle-haunch axis stimulation. Whatever the period of testing, the two groups never differed in terms of gregariousness or locomotor activity.

**Table 1:** Medians (interquartile) of the variables of personality as a function of treatment and test session (5, 12, or 23 weeks after the initiation of treatment).

Dimension and Variable measured	Week 5	Week 12	Week 23
**Fearfulness**			
Number of contacts with novel object	NS	M_EE_ = 8.5 (7.25–11.75) M_C_ = 4 (0–6) U = 18, *P* = 0.02	M_EE_ = 11.5 (8.25–16.5) M_C_ = 3 (0–9) U = 22.5, *P* = 0.05
Number of glances at novel object	M_EE_ = 2 (1.25–3.75) M_C_ = 9 (6–10) U = 12.5, *P* = 0.007	M_EE_ = 4 (2.25–4.75) M_C_ = 10 (8–13) U = 5, *P* = 0.001	M_EE_ = 1 (0.25–2.75) M_C_ = 8 (7–9) U = 4.5, *P* = 0.009
Latency to eat during novel area test (s)	NS	M_EE_ = 14.5 (11–19.5) M_C_ = 180 (180–180) U = 7.5, *P* = 0.001	M_EE_ = 23.5 (19.25–75.75) M_C_ = 180 (39–180) U = 16, *P* = 0.01
Latency to eat during suddenness test (s)	M_EE_ = 45 (33–160.25) M_C_ = 180 (140–180) U = 21.5, *P* = 0.04	NS	NS
**Gregariousness**			
Number of vocalizations during social isolation test	NS	NS	NS
**Locomotor activity**			
Number of sectors crossed	NS	NS	NS
**Tactile sensitivity**			
Response to von Frey filaments	NS	M_EE_ = 1 (1–1.5) M_C_ = 2.5 (2–3) U = 14, *P* = 0.01	NS
Reaction to stifle-haunch axis stimulation	M_EE_ = 2.18 (1.49–2.82) M_C_ = 5.5 (4–7.42) U = 16, *P* = 0.01	M_EE_ = 2.02 (1.56–2.87) M_C_ = 5.25 (3.75–5.74) U = 9, *P* = 0.003	M_EE_ = 1.37 (1.15–1.69) M_C_ = 2.67 (2.5–3.67) U = 12, *P* = 0.006
**Reactivity to humans**			
Number of contacts with passive human	M_EE_ = 10.5 (9.25–14.75) M_C_ = 2 (0–9) U = 14, *P* = 0.01	M_EE_ = 10 (8–12.75) M_C_ = 4 (0–4) U = 19.5, *P* = 0.03	NS
Latency to put on halter (s)	M_EE_ = 8 (8–8) M_C_ = 23 (13–37) U = 10, *P* = 0.001	M_EE_ = 8 (8–8) M_C_ = 12 (8–14) U = 15, *P* = 0.003	NS
Number of defensive reactions	M_EE_ = 0 (0–0) M_C_ = 4 (2–4) U = 1, *P* <0.0001	Not tested	Not tested

EE: EE-treated horses.

C: control horses.

U: Mann–Whitney U value.

NS: not significant.

### Learning tests

All the horses reached the shaping phase criterion in both groups (six successful trials on seven consecutive trials with each experimenter: A+ and B+; the plus means food reinforcement, minus means no reinforcement). As expected, the percentages of success did not differ significantly between the two experimenters (A+ and B+) in the two groups. During the Go/no-Go task, all the horses reached the criterion, but the EE-treated horses exhibited a higher percentage of success with experimenter A+ than with experimenter B−, who did not give food reinforcement. The median (interquartile) of the percentage of success with A+ was 88 (49–95) and with B− 65 (50–80) (Z = 2.12, *P* = 0.028), whereas no significant difference was found in the control horses. These results mean that in the Go/no-Go task, only the EE-treated horses behaved differently if we compared the experimenter who gave food reinforcement with the experimenter who did not give such reinforcement. In contrast, the control horses did not adapt their behavior during the Go/no-Go task.

### Salivary cortisol

**Figure 3:**
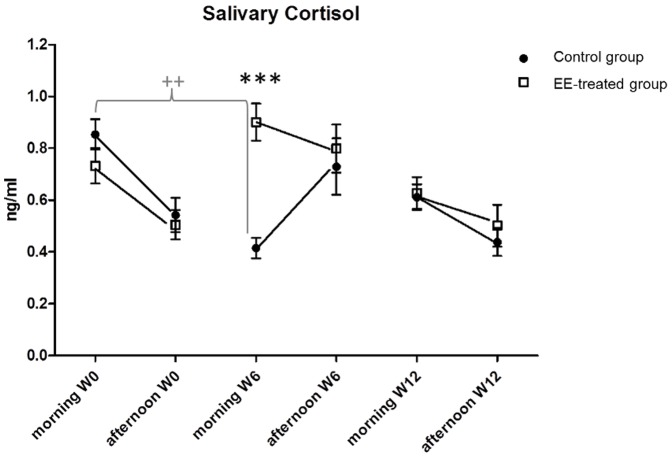
Salivary cortisol concentrations in the environmentally enriched and control groups. Salivary samples were collected 0, 6, and 12 weeks after the beginning of the treatment, in the morning (1000 h) or in the afternoon (1530 h). ***intergroup comparison, Mann–Whitney test, P≤0.001; ^++^intragroup comparison, Wilcoxon tests, P≤0.01.

Salivary cortisol concentrations were measured before treatment (week 0), in the middle of the treatment (after 6 weeks) and at the end of the treatment (after 12 weeks) in both groups (Figure 3). Intragroup comparison between week 0 and subsequent weeks showed significantly lower morning concentrations at the beginning of week 6 than at the beginning of week 0 in the control group (Z = −2.52, *P* = 0.011). Intergroup comparison indicated a significantly lower morning concentration in the control group than in the EE-treated group at the start of week 6 (U = 85, *P* = 0.001).

### The biological signature based on transcriptomic analysis

The expression of 43803 transcripts in blood cells from each animal of the two groups was assessed using gene expression microarrays. Using a stringent *P* value (<0.001), corresponding to a false discovery rate <5%, we identified 115 differentially expressed genes: 54 upregulated in the standard group and 61 upregulated in the EE-treated group. At *P*<0.005, the number of differentially expressed genes rose to 400: 155 upregulated genes in the Standard group and 245 upregulated genes in the EE-treated group. The lists of differentially expressed genes are provided in Tables S1 and S2.

**Figure 4:**
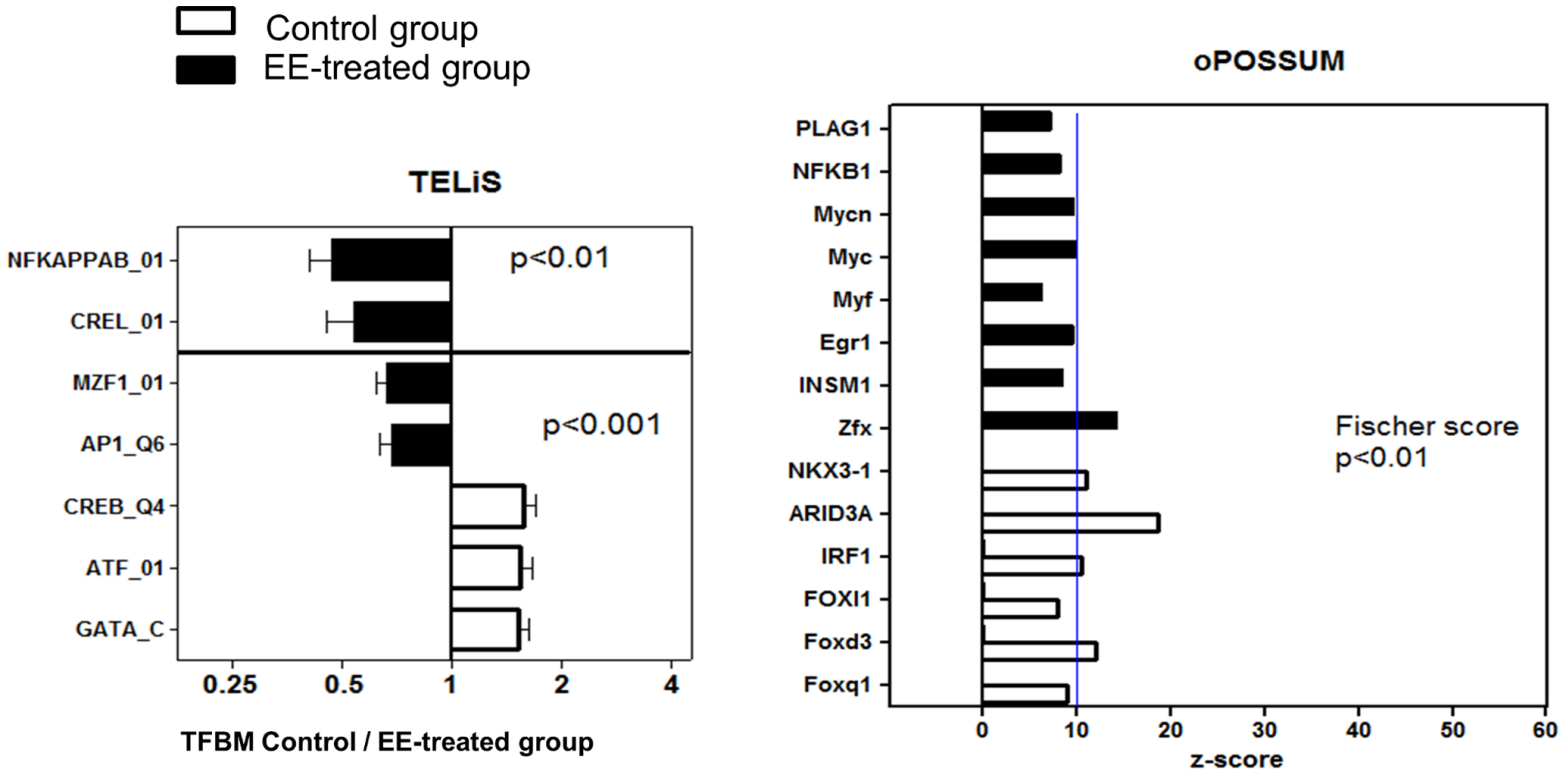
TELiS and oPOSSUM analyses of the genes differentially expressed between the environmentally enriched (EE) and the control groups. In TELiS analyses, the ratio of transcription factor-binding motif (TFBM) representation (control to EE horses) is shown. In the oPOSSUM analysis, significant over-represented transcription factors in either control or EE animals is shown using the resulting z-score. Differences in transcription factors with a z-score >10 and a Fisher score <0.01 are highly significant.

To examine the upstream signaling pathways that give rise to the transcriptional differences between the two groups, we first used TELiS bioinformatic analysis [28] on the 115 differentially expressed genes associated with *P*<0.001. This program highlights transcription factor-binding motifs that are over- or under-represented in a set of genes (e.g., genes encoding transcripts either upregulated or downregulated under specific conditions). Figure 4 shows a summary of the results obtained using the human JASPAR and TRANSFAC databases and the transcriptional shift analysis of TELiS. There is clear overrepresentation of GATA and CREB/ATF transcription factors in promoters of the genes upregulated in the control group and overrepresentation of the NF-κB family (c-REL, NFKB) and AP1 and MZF transcription factor motifs in the promoters of genes upregulated in the EE-treated animals. To extend this analysis, we used another bioinformatic software package, oPOSSUM [29], which analyzes the data in a different way (see File S1 for details). The binding motifs of the NF-κB family of transcription factors were found to be over-represented in the genes encoding transcripts that are upregulated in the EE-treated group (as in the TELiS analysis) along with other transcription factors such as Myc/Myf, Egr-1, INSM1, PLAG1, and Zfx. As for the control group, IRF1-binding sites were found to be over-represented, but CREB/ATF and GATA transcription factors were not detected at a significant level. Factors such as ARID3A, NKX3, and FoxD3, were activated more in the control group.

**Figure 5:**
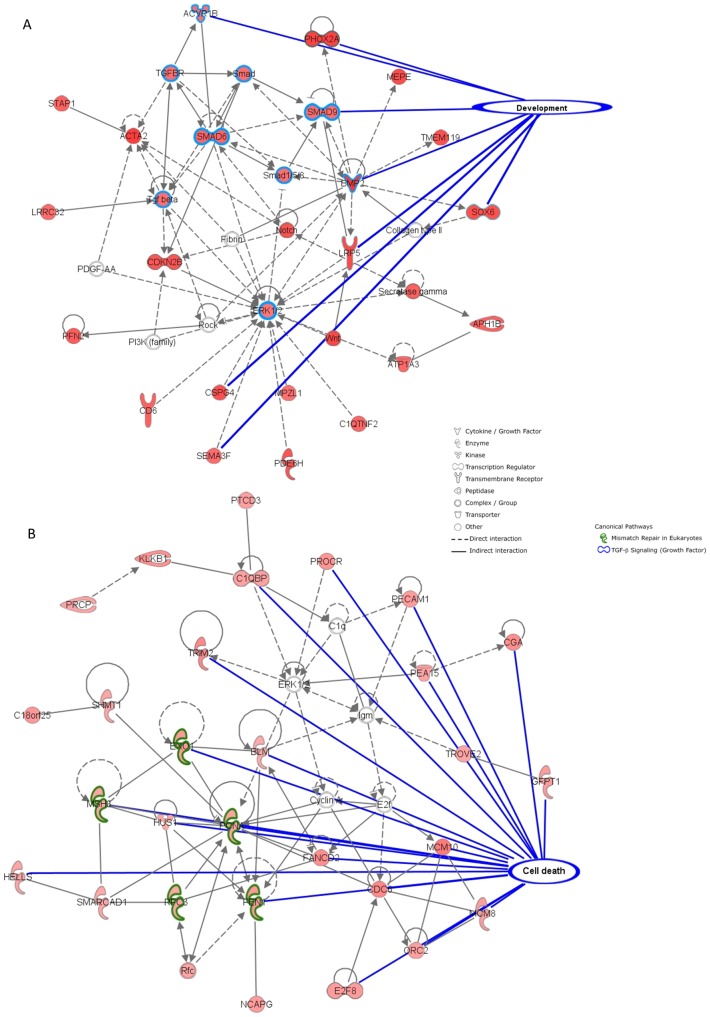
Illustration of the top gene network upregulated in the environmentally enriched (A) and control (B) groups. These data were generated by the Ingenuity Pathway Analysis (IPA) software using the 400 genes differentially expressed at *P*<0.005. The genes marked with a red symbol are from the list of differentially expressed genes, whereas the genes marked with a white symbol are intermediary genes of the network added by the software.

To gain insight into the function of the differentially expressed genes, we used the Ingenuity Pathway Analysis (IPA) software. In order to build significant networks, we used the set of 400 differentially expressed genes at *P*<0.005. The top score networks, biological functions, and canonical pathways calculated by the IPA software are presented in Tables S3 and S4. Figures 5a and 5b show an illustration of the top network in each group. The genes upregulated in the EE-treated group were predominately associated with cellular development (growth, proliferation, and differentiation: 81 molecules; Table S1), whereas the genes upregulated in the control group were associated with cell death (cell cycle: 43 molecules, mismatch repair: 35 molecules; Table S2).

## Discussion

This experiment revealed noticeable differences between the EE-treated group and the control group at the behavioral level, with an impact on well-being, personality, and learning as well as on the gene expression profile of blood cells. One can see a distinct pattern of effects (or a “fingerprint”) of EE on the individual.

### The behavioral fingerprint of the Enriched Environment

EE had strong effects on well-being, confirming the findings in other species [6], [7]. From the first days, EE-treated horses vocalize and exhibit alert postures and aberrant behavioral patterns less often. They are found lying down more often. From the third week, EE-treated horses displayed ears pointed backwards less often. Furthermore, eating problems were present in four out of the nine control horses, but there were none in the EE-treated group. All these indicators point to a lower level of perceived stress and better well-being in EE-treated horses [22], [30], [31]. Each type of the enrichment involved in this experiment could by itself improve well-being. For instance, more lying down among the EE-treated animals may be due to the larger size of their stall, to the straw bedding, or to the appeasing effect of the music [25], [32], [33]. The lower frequency of aberrant behavioral patterns may be due to the access to pasture with conspecific animals or to the feeding distribution and composition [22], [24], [34]. Nevertheless, according to the studies that have compared the effect of one type of enrichment at a time versus a combination of several of types of enrichment, it is highly probable that it is the combination of all these types of enrichment that lead to the numerous beneficial effects [1], [35].

EE also clearly and durably modifies the personality of horses, particularly the dimensions of fearfulness, tactile sensitivity, and reactivity to humans. In particular, EE-treated horses are less frightened by suddenness and novelty (objects or a novel area), in line with another study, which reported a decrease in anxiety after EE [1]. Nonetheless, EE also attenuates avoidance reactions toward tactile stimuli, human presence, and handling. Thus, these changes reflect broader effects than the classic effects on anxiety. Taken together, these observations seem to demonstrate a more positive perception of the environment, whether it concerns potentially frightening stimuli (novel or sudden) or nonfrightening ones (harmless tactile stimuli) or the human presence. In contrast, control horses perceive their environment as more threatening and attempt to avoid these stimuli. The finding that EE induces more positive affective states is in line with experiments on EE involving a sophisticated cognitive bias paradigm [36]–[39]. In addition, EE-treated horses appear to be more prone to interaction with a novel object and with passive humans, suggesting that these horses have a higher level of curiosity. Surprisingly, when the two groups of horses return to the same pasture, the effects on fearfulness and sensitivity persist for at least 3 months after the end of the treatment. Although personality is reported to be stable when horses are continuously housed in the same environment [11]–[14], we show here that a drastic change of environment, such as EE at a young age, can modify personality for an extended period of time. This experiment contributes to a better understanding of the influence of the environment on the development of personality.

Finally, with respect to learning abilities, EE-treated horses show better performance in the Go/No-Go task. The finding that the two groups differ only in learning the Go/No-Go task but not in the shaping phase suggests that this difference is not due to a higher motivation to obtain a reward but rather to a better ability of the EE-treated horses to solve a complex cognitive task that requires more focused attention [40]. This better ability could be related to the change of their personality. Indeed, a relationship between a low level of fearfulness and high performance in instrumental learning has been described [16], [41] and is explained by the better focus of attention on the task. EE-treated horses thus appear to be more attentive and more prone to detect the cues given by humans in the Go/No-Go task; these effects can explain the better performance.

### The biological fingerprint of the Enriched Environment

EE appears to influence the cortisol level temporarily. At the start of week 6, control horses exhibit lower morning cortisol concentration than do enriched horses. A reduced cortisol level has already been reported in horses living in conditions similar to our control treatment (i.e., isolated in an individual stall without any additional sensory stimulation), in particular, in horses that display behavioral despair [30], [42]. In other social mammals, chronic isolation leads to either elevated, lower, or unchanged basal levels of glucocorticoids depending on age, duration of isolation, and the species [43]. Similarly, in the literature, there is no consensus on the effects of EE on corticosterone/cortisol level, and all the possible outcomes have been reported: an increase, decrease, or no change [1], [4]. We not only observed a decrease in the morning level of cortisol but also an increase in afternoon concentration, although the difference is not statistically significant. This result points to inversion of the circadian rhythm of cortisol in control horses at the beginning of week 6, which is very interesting in regards to the importance of the circadian rhythm of cortisol secretion for preparing and maintaining the brain and body in an optimal state [44]. Nevertheless, this effect on cortisol concentration was only temporary because it disappeared at the beginning of week 12. This phenomenon may be explained by the fact that between weeks 6 and 12, the horses were exposed to behavioral tests that constitute a kind of enrichment for the horses in control conditions; thus, this intervention restored the cortisol secretion.

The second result concerns the transcriptomic fingerprint of EE. The availability of a horse-specific microarray and of the horse genome sequence has opened up the opportunities for further research into the biological impact of EE on horses. By coupling gene expression data with several bioinformatic analyses, we can show that EE and control conditions have divergent effects on gene expression in blood cells. According to the TELiS analyses, there is pronounced activation of CREB/ATF and GATA families of transcription factors in the control group. In the EE-treated horses, there was a tendency for upregulation of target gens of NF-κB (NF-κB and REL factors), MZF, and AP1. Overrepresentation of the CREB transcription factors in chronically isolated versus socially integrated people has been observed previously [45]. In another study, β-adrenergic activation of the GATA1 transcription factor was found in mice subjected to a social threat; a similar finding was reported for human depressive subjects (compared to control) [46]. Because CREB/ATF transcription factors convey adrenergic signals to the transcriptome in blood cells, it is possible that the activation of CREB/ATF and GATA transcription factors in the control group of horses might reflect the poor social interactions associated with the low learning performance and high levels of fearfulness in these horses. According to the oPOSSUM analysis, overrepresentation of transcription factors other than CREB/ATF and GATA was detected in the control horses; only the NF-κB-related findings were common between the two types of analysis. In human social genomic studies, IRF-1 is usually found to be downregulated in isolated/stressed individuals, and this phenomenon is thought to reflect an adaptive reaction to the low risk of dissemination of a viral infection in isolated individuals [47]. Here, this hypothesis is not supported by our data because control animals are isolated most of the time but display activation of IRF-1. Rather, activation of IRF-1 in the control group supports the notion that interferon induces a loss of appetite and sickness-like behavior because 4 out of 9 animals in the control group lost their appetite at the end of the experiment. A vet evaluated the animals with eating problems for infectious disease using standard clinical metrics (mostly core body temperature) and found no evidence of an active infection that might explain the observed transcriptomic alterations in immune cells. The oPOSSUM analysis showed overrepresentation of such factors as ARID3, NKX3-1, Foxq1, and FoxD3 (in addition to IRF-1) which are all involved in cell cycle regulation. Finally, IPA revealed that in the control group, a network of genes is upregulated that is involved in mismatch repair and cell death-related processes.

Regarding the animals exposed to EE, both the TELiS and oPOSSUM analyses show activation of the NF-κB family of transcription factors. The upregulation of the genes controlled by these transcription factors may be linked to greater amounts of physical exercise performed by the EE-treated horses or may reflect more frequent exposure to bacteria because these horses spend more time outdoors than do the control animals. Nonetheless, proinflammatory genes are not activated noticeably in these animals. Rather, factors with a known anti-inflammatory role such as PPARγ/RXRα [48] are also activated in the EE group. Additionally, upregulation of other transcription factors in the EE group, such as the Myc/Myf family, Egr-1, INSM1, and Zfx, as well as the results of IPA point to activation of functions related to proliferation and differentiation of blood cells. These functions in young horses probably reflect a healthy state of development of the organism. Whether the increased cell proliferation and differentiation also occur in the brain (meaning increased neurogenesis in the EE-treated horses) was not explored in this study but would be an interesting question for a future project in light of the improved well-being and learning abilities of the EE-treated animals. Egr-1, for example, would be a good candidate because it is involved not only in differentiation and activation of immune cells [49] but also in neuronal plasticity [50]. Accordingly, INSM1 also performs a function in early embryonic neurogenesis [51].

## Conclusion

Overall, our data show that a 12-week EE combining complex inanimate and social stimuli has beneficial effects on horses in terms of personality traits, learning abilities, and general well-being. The observed effects of EE (versus control) on the transcriptome of blood cells confirm a more favorable biological profile of EE-treated horses. This study appears to be the first to report both behavioral and molecular effects of EE on horses. In addition to demonstrating interesting properties of EE, these results support the use of EE for improvement of the well-being of horses as well as security of the handlers.

## Supporting Information

Table S1 **List of differentially regulated gene associated with p<0.001.**
(DOCX)

Table S2 **List of differentially regulated gene associated with p<0.005.**
(DOCX)

Table S3 **IPA core analysis, direct and indirect: Enriched (EE-treated) group.**
(DOCX)

Table S4 **IPA core analysis, direct and indirect: Control group.**
(DOCX)

File S1 **Supplementary methods.**
(DOCX)

Appendix S1 **Personality data.**
(PDF)

Appendix S2 **Learning data.**
(PDF)

Appendix S3 **Cortisol data.**
(PDF)

## References

[1] Fox C, Merali Z, Harrison C (2006) Therapeutic and protective effect of environmental enrichment against psychogenic and neurogenic stress. Behav Brain Res 175:1–8. 16970997 10.1016/j.bbr.2006.08.016

[2] Bekinschtein P, Oomen CA, Saksida LM, Bussey TJ (2011) Effects of environmental enrichment and voluntary exercise on neurogenesis, learning and memory, and pattern separation: BDNF as a critical variable? Semin Cell Dev Biol 22:536–542. 21767656 10.1016/j.semcdb.2011.07.002

[3] Cotman CW, Berchtold NC (2007) Physical activity and the maintenance of cognition: Learning from animal models. Alzheimer's & Dementia 3:S30–S37. 10.1016/j.jalz.2007.01.013 19595972

[4] Simpson J, Kelly JP (2011) The impact of environmental enrichment in laboratory rats—Behavioural and neurochemical aspects. Behav Brain Res 222:246–264. 21504762 10.1016/j.bbr.2011.04.002

[5] Petrosini L, De Bartolo P, Foti F, Gelfo F, Cutuli D, et al (2009) On whether the environmental enrichment may provide cognitive and brain reserves. Brain Res Rev 61:221–239. 19631687 10.1016/j.brainresrev.2009.07.002

[6] Mason G, Clubb R, Latham N, Vickery S (2007) Why and how should we use environmental enrichment to tackle stereotypic behaviour? Appl Anim Behav Sci 102:163–188.

[7] Wells DL (2009) Sensory stimulation as environmental enrichment for captive animals: A review. Appl Anim Behav Sci 118:1–11.

[8] Claxton AM (2011) The potential of the human–animal relationship as an environmental enrichment for the welfare of zoo-housed animals. Appl Anim Behav Sci 133:1–10.

[9] Bates JE (1989) Concepts and measures of temperament. In: Kohnstamm GA, Bates JE, Rothbart MKeditors. Temperament in childhood. New-York: Wiley. pp.3–26.

[10] Borstel UKv (2013) Assessing and influencing personality for improvement of animal welfare: a review of equine studies. CAB reviews 8:1–27.

[11] Lansade L, Bouissou M-F (2008) Reactivity to humans: A temperament trait of horses which is stable across time and situations. Appl Anim Behav Sci 114:492–508.

[12] Lansade L, Bouissou M-F, Erhard HW (2008) Fearfulness in horses: A temperament trait stable across time and situations. Appl Anim Behav Sci 115:182–200.

[13] Lansade L, Bouissou M-F, Erhard HW (2008) Reactivity to isolation and association with conspecifics: A temperament trait stable across time and situations. Appl Anim Behav Sci 109:355–373.

[14] Lansade L, Pichard G, Leconte M (2008) Sensory sensitivities: Components of a horse's temperament dimension. Appl Anim Behav Sci 114:534–553.

[15] Valenchon M, Lévy F, Fortin M, Leterrier C, Lansade L (2013) Stress and temperament affect working memory performance for disappearing food in horses, Equus caballus. Anim Behav 86:1233–1240.

[16] Valenchon M, Levy F, Prunier A, Moussu C, Calandreau L, et al (2013) Stress Modulates Instrumental Learning Performances in Horses (Equus caballus) in Interaction with Temperament. PLoS ONE 8. 10.1371/journal.pone.0062324 PMC3633893 23626801

[17] Slavich GM, Cole SW (2013) The Emerging Field of Human Social Genomics. Clin Psychol Sci 1:331–348. 23853742 10.1177/2167702613478594 PMC3707393

[18] Cole SW (2013) Social Regulation of Human Gene Expression: Mechanisms and Implications for Public Health. Am J Public Health 103:84–92. 10.2105/AJPH.2012.301183 PMC3786751 23927506

[19] Robinson GE, Fernald RD, Clayton DF (2008) Genes and Social Behavior. Science 322:896–900. 18988841 10.1126/science.1159277 PMC3052688

[20] Waters AJ, Nicol CJ, French NP (2002) Factors influencing the development of stereotypic and redirected behaviours in young horses: findings of a four year prospective epidemiological study. Equ Vet J 34:572–579. 10.2746/042516402776180241 12357996

[21] Rivera E, Benjamin S, Nielsen B, Shelle J, Zanella AJ (2002) Behavioral and physiological responses of horses to initial training: the comparison between pastured versus stalled horses. Appl Anim Behav Sci 78:235–252.

[22] Heleski CR, Shelle AC, Nielsen BD, Zanella AJ (2002) Influence of housing on weanling horse behavior and subsequent welfare. Appl Anim Behav Sci 78:291–302.

[23] Søndergaard E, Ladewig J (2004) Group housing exerts a positive effect on the behaviour of young horses during training. Appl Anim Behav Sci 87:105–118.

[24] Goodwin D, Davidson HPB, Harris P (2005) Sensory varieties in concentrate diets for stabled horses: effects on behaviour and selection. Appl Anim Behav Sci 90:337–349.

[25] Houpt K, Marrow M, Seeliger M (2000) A preliminary study of the effect of music on Equine behavior. J Vet Sci 11:691–737.

[26] Jorgensen GHM, Liestol SH-O, Boe KE (2011) Effects of enrichment items on activity and social interactions in domestic horses (Equus caballus). Appl Anim Behav Sci 129:100–110.

[27] Barrey E, Mucher E, Jeansoule N, Larcher T, Guigand L, et al (2009) Gene expression profiling in equine polysaccharide storage myopathy revealed inflammation, glycogenesis inhibition, hypoxia and mitochondrial dysfunctions. BMC Vet Res 5:29. 19664222 10.1186/1746-6148-5-29 PMC2741442

[28] Cole SW, Yan W, Galic Z, Arevalo J, Zack JA (2005) Expression-based monitoring of transcription factor activity: the TELiS database. Bioinformatics 21:803–810. 15374858 10.1093/bioinformatics/bti038

[29] Kwon AT, Arenillas DJ, Hunt RW, Wasserman WW (2012) oPOSSUM-3: Advanced Analysis of Regulatory Motif Over-Representation Across Genes or ChIP-Seq Datasets. G3-Genes Genomes Genetics 2:987–1002. 10.1534/g3.112.003202 PMC3429929 22973536

[30] Fureix C, Jego P, Henry S, Lansade L, Hausberger M (2012) Towards an Ethological Animal Model of Depression? A Study on Horses. PLoS ONE 7. 10.1371/journal.pone.0039280 PMC3386251 22761752

[31] Young T, Creighton E, Smith T, Hosie C (2012) A novel scale of behavioural indicators of stress for use with domestic horses. Appl Anim Behav Sci 140:33–43.

[32] Pedersen GR, Søndergaard E, Ladewig J (2004) The influence of bedding on the time horses spend recumbent. J Equ Vet Sci 24:153–158.

[33] Raabymagle P, Ladewig J (2006) Lying behavior in horses in relation to box size. J Equ Vet Sci 26:11–17.

[34] Goodwin D, Davidson HPB, Harris P (2007) Responses of horses offered a choice between stables containing single or multiple forages. Vet Rec. 160 548 17449710 10.1136/vr.160.16.548

[35] van Praag H, Kempermann G, Gage FH (2000) Neural consequences of environmental enrichment. Nat Rev Neurosci 1:191–198. 11257907 10.1038/35044558

[36] Brydges NM, Leach M, Nicol K, Wright R, Bateson M (2011) Environmental enrichment induces optimistic cognitive bias in rats. Anim Behav 81:169–175.

[37] Douglas C, Bateson M, Walsh C, Bédué A, Edwards SA (2012) Environmental enrichment induces optimistic cognitive biases in pigs. Appl Anim Behav Sci 139:65–73.

[38] Matheson SM, Asher L, Bateson M (2008) Larger, enriched cages are associated with ‘optimistic’ response biases in captive European starlings (Sturnus vulgaris). Appl Anim Behav Sci 109:374–383.

[39] Richter SH, Schick A, Hoyer C, Lankisch K, Gass P, et al (2012) A glass full of optimism: Enrichment effects on cognitive bias in a rat model of depression. Cognitive Affective & Behavioral Neuroscience 12:527–542. 10.3758/s13415-012-0101-2 22644760

[40] Criaud M, Boulinguez P (2013) Have we been asking the right questions when assessing response inhibition in go/no-go tasks with fMRI? A meta-analysis and critical review. Neurosci Biobehav Rev 37:11–23. 23164813 10.1016/j.neubiorev.2012.11.003

[41] Lansade L, Coutureau E, Marchand A, Baranger G, Valenchon M, et al**.** (2013) Dimensions of Temperament Modulate Cue-Controlled Behavior: A Study on Pavlovian to Instrumental Transfer in Horses (Equus Caballus). PLoS ONE 8. 10.1371/journal.pone.0064853 PMC3682987 23798994

[42] Visser EK, Ellis AD, Van Reenen CG (2008) The effect of two different housing conditions on the welfare of young horses stabled for the first time. Appl Anim Behav Sci 114:521–533.

[43] Hawkley LC, Cole SW, Capitanio JP, Norman GJ, Cacioppo JT (2012) Effects of social isolation on glucocorticoid regulation in social mammals. Horm Behav 62:314–323. 22663934 10.1016/j.yhbeh.2012.05.011 PMC3449017

[44] Lightman SL, Conway-Campbell BL (2010) The crucial role of pulsatile activity of the HPA axis for continuous dynamic equilibration. Nat Rev Neurosci 11:710–718. 20842176 10.1038/nrn2914

[45] Cole SW, Hawkley LC, Arevalo JM, Sung CY, Rose RM, et al (2007) Social regulation of gene expression in human leukocytes. Genome Biol 8. 10.1186/gb-2007-8-9-r189 PMC2375027 17854483

[46] Cole SW, Arevalo JMG, Takahashi R, Sloan EK, Lutgendorf SK, et al (2010) Computational identification of gene-social environment interaction at the human IL6 locus. Proc Natl Acad Sci U S A 107:5681–5686. 20176930 10.1073/pnas.0911515107 PMC2851818

[47] Eisenberger NI, Cole SW (2012) Social neuroscience and health: neurophysiological mechanisms linking social ties with physical health. Nat Neurosci 15:669–674. 22504347 10.1038/nn.3086

[48] Delerive P, Fruchart JC, Staels B (2001) Peroxisome proliferator-activated receptors in inflammation control. J Endocrinol 169:453–459. 11375115 10.1677/joe.0.1690453

[49] Collins S, Lutz MA, Zarek PE, Anders RA, Kersh GJ, et al (2008) Opposing regulation of T cell function by Egr-1/NAB2 and Egr-2/Egr-3. Eur J Immunol 38:528–536. 18203138 10.1002/eji.200737157 PMC3598016

[50] O'Donovan KJ, Tourtellotte WG, Milbrandt J, Baraban JM (1999) The EGR family of transcription-regulatory factors: progress at the interface of molecular and systems neuroscience. Trends Neurosci 22:167–173. 10203854 10.1016/s0166-2236(98)01343-5

[51] Lan MS, Breslin MB (2009) Structure, expression, and biological function of INSM1 transcription factor in neuroendocrine differentiation. FASEB J 23:2024–2033. 19246490 10.1096/fj.08-125971 PMC2704596

