# Supplementary material for: Behavioral and Transcriptomic Fingerprints of an Enriched Environment in Horses (Equus caballus)
Source: PLoS One. 2014 Dec 10;9(12):e114384. doi: 10.1371/journal.pone.0114384 (PMC4262392; doi:10.1371/journal.pone.0114384)
Supplement: Table S1 — List of differentially regulated gene associated with p<0.001. (DOCX) [file pone.0114384.s001.docx]

**Table S1**: list of differentially regulated gene associated with p <0.001

| Probe Set ID | *p*-value | Gene symbol | Fold change (Enriched/Control) |
| --- | --- | --- | --- |
| A_69_P001552 | 3,78E-04 | FCER2 | 0,50 |
| A_69_P039091 | 2,30E-04 | CRIP3 | 0,54 |
| A_69_P061576 | 3,85E-05 | CCNJL | 0,55 |
| A_69_P030367 | 8,45E-04 | PCYT1A | 0,56 |
| A_69_P019961 | 9,07E-04 | RPL3L | 0,59 |
| A_69_P082341 | 5,40E-04 | KCNG4 | 0,60 |
| A_69_P036106 | 9,99E-04 | C17orf76 | 0,61 |
| A_69_P025906 | 7,75E-04 | CASS4 | 0,62 |
| A_69_P076192 | 4,02E-04 | BCL9 | 0,63 |
| A_69_P097651 | 1,13E-04 | CSPG4 | 0,63 |
| A_69_P122581 | 9,76E-04 | SOX6 | 0,63 |
| A_69_P042368 | 7,03E-04 | HOXD3 | 0,64 |
| A_69_P045301 | 2,48E-04 | LRP3 | 0,64 |
| A_69_P052171 | 9,94E-04 | PDE6H | 0,64 |
| A_69_P065216 | 1,04E-05 | TESC | 0,64 |
| Oligo-375 | 9,66E-04 | UCP2 | 0,64 |
| A_69_P021751 | 5,98E-04 | TSPO | 0,65 |
| Oligo-245 | 8,14E-04 | MRPS7 | 0,66 |
| A_69_P018566 | 4,37E-05 | ZNF771 | 0,66 |
| A_69_P049951 | 4,16E-04 | SGK1 | 0,67 |
| A_69_P007006 | 2,63E-04 | C19orf20 | 0,68 |
| A_69_P096276 | 6,25E-04 | PCNXL2 | 0,68 |
| A_69_P087887 | 2,21E-04 | CCDC27 | 0,69 |
| A_69_P116086 | 5,73E-04 | NPDC1 | 0,69 |
| A_69_P031936 | 5,41E-04 | RECQL5 | 0,69 |
| A_69_P031896 | 4,42E-04 | TRIM47 | 0,69 |
| A_69_P074766 | 1,10E-04 | CCDC19 | 0,70 |
| A_69_P067811 | 9,04E-04 | TRAPPC5 | 0,70 |
| A_69_P064651 | 3,53E-05 | GATSL3 | 0,71 |
| A_69_P055731 | 5,43E-04 | PCBP4 | 0,71 |
| A_69_P003891 | 2,59E-04 | SMAD6 | 0,71 |
| A_69_P099056 | 3,70E-04 | C15orf52 | 0,72 |
| A_69_P089846 | 2,53E-05 | ASMT | 0,73 |
| A_69_P028857 | 9,21E-04 | EGFLAM | 0,73 |
| A_69_P004861 | 8,16E-04 | OXT | 0,73 |
| A_69_P125576 | 3,42E-04 | PRDM16 | 0,73 |
| A_69_P021191 | 9,78E-04 | TST | 0,73 |
| A_69_P037756 | 5,77E-04 | ZFP57 | 0,73 |
| A_69_P086377 | 9,17E-05 | CCDC28B | 0,74 |
| A_69_P021601 | 7,69E-04 | CSDC2 | 0,74 |
| A_69_P117200 | 6,27E-04 | RAVER1 | 0,74 |
| A_69_P054361 | 1,61E-04 | MARCH9 | 0,75 |
| A_69_P086366 | 7,95E-04 | DCDC2B | 0,75 |
| A_69_P044632 | 2,12E-04 | SQLE | 0,75 |
| A_69_P086966 | 4,42E-04 | LYPLA2 | 0,76 |
| A_69_P086331 | 3,19E-04 | MARCKSL1 | 0,76 |
| A_69_P129476 | 3,89E-04 | TPM1 | 0,76 |
| A_69_P000841 | 7,88E-04 | COMT | 0,77 |
| A_69_P105476 | 7,39E-04 | MPZL1 | 0,77 |
| A_69_P051546 | 2,56E-04 | TAPBPL | 0,77 |
| A_69_P010670 | 3,36E-04 | TMEM179B | 0,77 |
| A_69_P085566 | 5,45E-04 | B4GALT2 | 0,78 |
| A_69_P076181 | 9,91E-04 | GJA5 | 0,78 |
| A_69_P037831 | 1,00E-04 | GNL1 | 0,78 |
| A_69_P018696 | 4,01E-04 | PPP4C | 0,78 |
| A_69_P051271 | 8,27E-04 | FBXL14 | 0,79 |
| A_69_P074761 | 7,54E-04 | TAGLN2 | 0,79 |
| A_69_P020386 | 5,46E-04 | GLIPR1 | 0,80 |
| A_69_P061131 | 5,15E-04 | GRK6 | 0,80 |
| A_69_P018716 | 1,87E-04 | MAPK3 | 0,80 |
| A_69_P011626 | 3,98E-04 | TSPAN32 | 0,80 |
| A_69_P096911 | 2,26E-04 | BLM | 1,20 |
| A_69_P061192 | 1,15E-04 | UIMC1 | 1,20 |
| A_69_P078306 | 6,68E-04 | HUS1 | 1,21 |
| A_69_P012687 | 8,17E-04 | PITRM1 | 1,21 |
| A_69_P083851 | 1,27E-04 | SLAIN2 | 1,21 |
| A_69_P107357 | 1,23E-04 | DDX18 | 1,22 |
| A_69_P079571 | 7,53E-04 | ING3 | 1,22 |
| A_69_P020771 | 3,70E-04 | NEDD1 | 1,22 |
| A_69_P057244 | 3,03E-04 | TOPBP1 | 1,23 |
| A_69_P015508 | 9,47E-04 | ZFP36L2 | 1,25 |
| A_69_P036846 | 7,41E-04 | CDKAL1 | 1,26 |
| A_69_P034363 | 1,03E-05 | HEATR6 | 1,26 |
| A_69_P095787 | 5,01E-04 | DNAJC9 | 1,27 |
| A_69_P086161 | 5,90E-06 | SFPQ | 1,27 |
| A_69_P063686 | 8,41E-05 | GFM2 | 1,28 |
| A_69_P095051 | 3,56E-04 | HELLS | 1,28 |
| A_69_P070936 | 9,85E-05 | PRCP | 1,29 |
| A_69_P058957 | 3,11E-04 | MOBKL1B | 1,31 |
| A_69_P074221 | 3,31E-04 | INTS7 | 1,32 |
| Oligo-281 | 8,64E-04 | PEA15 | 1,32 |
| A_69_P078961 | 7,97E-04 | SNX13 | 1,33 |
| A_69_P035436 | 8,29E-05 | C1QBP | 1,34 |
| A_69_P022596 | 4,14E-04 | RAD51B | 1,34 |
| A_69_P129091 | 8,84E-04 | AHNAK | 1,35 |
| A_69_P058661 | 2,45E-04 | PTCD3 | 1,36 |
| A_69_P096028 | 1,88E-04 | C1orf96 | 1,38 |
| A_69_P024282 | 6,52E-04 | MCM8 | 1,38 |
| A_69_P059206 | 4,33E-05 | GFPT1 | 1,39 |
| A_69_P107546 | 7,80E-04 | NRP2 | 1,39 |
| A_69_P074202 | 6,88E-04 | TMEM206 | 1,39 |
| A_69_P083967 | 9,82E-04 | ATP8A1 | 1,40 |
| A_69_P089041 | 3,62E-04 | PET112 | 1,42 |
| A_69_P032988 | 6,53E-04 | PSMC3IP | 1,43 |
| A_69_P116114 | 3,18E-04 | C18orf25 | 1,45 |
| A_69_P044201 | 4,60E-04 | RGS22 | 1,45 |
| A_69_P042851 | 8,19E-04 | SGOL2 | 1,45 |
| A_69_P040417 | 6,08E-04 | ZC3H13 | 1,45 |
| A_69_P122867 | 9,68E-04 | ACYP1 | 1,46 |
| A_69_P059652 | 9,41E-04 | MCFD2 | 1,47 |
| A_69_P096467 | 7,84E-04 | ERCC6 | 1,48 |
| A_69_P039761 | 8,83E-04 | SKA3 | 1,52 |
| A_69_P024717 | 6,42E-04 | TPX2 | 1,55 |
| A_69_P113095 | 1,54E-04 | KIAA1671 | 1,56 |
| A_69_P056886 | 7,01E-04 | OSBPL10 | 1,58 |
| A_69_P122131 | 8,14E-04 | DDX60L | 1,59 |
| A_69_P013262 | 8,05E-04 | SMC2 | 1,59 |
| A_69_P061679 | 4,64E-05 | HAVCR1 | 1,65 |
| A_69_P033342 | 8,22E-04 | CDC6 | 1,68 |
| A_69_P013427 | 1,20E-04 | CTNNAL1 | 1,75 |
| A_69_P012446 | 7,98E-04 | MCM10 | 1,88 |
| A_69_P026876 | 7,89E-05 | VLDLR | 1,91 |
| A_69_P008332 | 2,37E-04 | ENAH | 1,96 |
| A_69_P080056 | 3,03E-04 | ATP6V0A4 | 1,97 |
| A_69_P029546 | 1,00E-05 | SERPINI1 | 2,05 |
