# Supplementary material for: Behavioral and Transcriptomic Fingerprints of an Enriched Environment in Horses (Equus caballus)
Source: PLoS One. 2014 Dec 10;9(12):e114384. doi: 10.1371/journal.pone.0114384 (PMC4262392; doi:10.1371/journal.pone.0114384)
Supplement: Table S2 — List of differentially regulated gene associated with p<0.005. (DOCX) [file pone.0114384.s002.docx]

**Table S2:** list of differentially regulated gene associated with p <0.005

| Probe Set ID | *p*-value | Gene symbol | Fold change (enriched/control) |
| --- | --- | --- | --- |
| A_69_P001551 | 1,66E-03 | FCER2 | 0,52 |
| A_69_P039091 | 2,30E-04 | CRIP3 | 0,54 |
| A_69_P002111 | 2,47E-03 | BMP2 | 0,55 |
| A_69_P061576 | 3,85E-05 | CCNJL | 0,55 |
| A_69_P038871 | 2,11E-03 | TREM2 | 0,55 |
| A_69_P030367 | 8,45E-04 | PCYT1A | 0,56 |
| A_69_P123477 | 4,46E-03 | PRICKLE2 | 0,56 |
| A_69_P003771 | 3,75E-03 | ACTA2 | 0,57 |
| A_69_P052283 | 4,76E-03 | C12orf39 | 0,57 |
| A_69_P090946 | 1,92E-03 | PCSK1N | 0,57 |
| A_69_P075622 | 4,61E-03 | S100A12 | 0,57 |
| A_69_P062571 | 3,01E-03 | C5orf20 | 0,58 |
| A_69_P110788 | 3,04E-03 | MTMR2 | 0,58 |
| A_69_P071316 | 2,64E-03 | PHOX2A | 0,58 |
| A_69_P006941 | 1,62E-03 | SGTA | 0,58 |
| A_69_P072532 | 2,10E-03 | ST5 | 0,58 |
| A_69_P019961 | 9,07E-04 | RPL3L | 0,59 |
| A_69_P082341 | 5,40E-04 | KCNG4 | 0,60 |
| A_69_P033821 | 3,55E-03 | SAMD14 | 0,60 |
| A_69_P111961 | 1,03E-03 | UBP1 | 0,60 |
| A_69_P032579 | 4,13E-03 | WNT3 | 0,60 |
| A_69_P036106 | 9,99E-04 | C17orf76 | 0,61 |
| A_69_P123031 | 1,65E-03 | COX7A1 | 0,61 |
| A_69_P033686 | 3,14E-03 | HOXB6 | 0,61 |
| A_69_P049446 | 1,25E-03 | LAMA4 | 0,61 |
| A_69_P011501 | 1,92E-03 | LRP5 | 0,61 |
| A_69_P082976 | 1,74E-03 | MEPE | 0,61 |
| A_69_P087981 | 3,31E-03 | TMEM52 | 0,61 |
| A_69_P021101 | 1,19E-03 | TOM1 | 0,61 |
| A_69_P025906 | 7,75E-04 | CASS4 | 0,62 |
| A_69_P007246 | 2,69E-03 | FGD1 | 0,62 |
| A_69_P114409 | 4,92E-03 | HAND1 | 0,62 |
| Oligo-257 | 2,84E-03 | MT-ND2 | 0,62 |
| A_69_P059176 | 3,15E-03 | SNRNP27 | 0,62 |
| A_69_P076192 | 4,02E-04 | BCL9 | 0,63 |
| A_69_P031366 | 1,94E-03 | CCDC57 | 0,63 |
| A_69_P097651 | 1,13E-04 | CSPG4 | 0,63 |
| A_69_P122581 | 9,76E-04 | SOX6 | 0,63 |
| A_69_P064986 | 4,48E-03 | TMEM119 | 0,63 |
| A_69_P060436 | 4,00E-03 | FKBP1B | 0,64 |
| A_69_P042368 | 7,03E-04 | HOXD3 | 0,64 |
| A_69_P106133 | 1,36E-03 | KDELR3 | 0,64 |
| A_69_P049141 | 2,08E-03 | LIN28B | 0,64 |
| A_69_P045301 | 2,48E-04 | LRP3 | 0,64 |
| A_69_P014871 | 3,03E-03 | NOTCH1 | 0,64 |
| A_69_P052171 | 9,94E-04 | PDE6H | 0,64 |
| A_69_P108736 | 4,25E-03 | SPEN | 0,64 |
| A_69_P065216 | 1,04E-05 | TESC | 0,64 |
| Oligo-375 | 9,66E-04 | UCP2 | 0,64 |
| A_69_P066107 | 1,92E-03 | ANKRD29 | 0,65 |
| A_69_P004871 | 3,48E-03 | CDKN2B | 0,65 |
| A_69_P084636 | 3,08E-03 | DOK7 | 0,65 |
| A_69_P059049 | 2,74E-03 | EMX1 | 0,65 |
| A_69_P054054 | 3,64E-03 | IKZF4 | 0,65 |
| A_69_P023551 | 2,84E-03 | SLC25A47 | 0,65 |
| A_69_P014751 | 3,27E-03 | SURF6 | 0,65 |
| A_69_P021751 | 5,98E-04 | TSPO | 0,65 |
| A_69_P110551 | 2,42E-03 | A1BG | 0,66 |
| A_69_P034726 | 1,09E-03 | ALDOC | 0,66 |
| A_69_P016802 | 3,64E-03 | B3GALT5 | 0,66 |
| A_69_P071101 | 4,03E-03 | GDPD5 | 0,66 |
| A_69_P033331 | 4,58E-03 | GJD3 | 0,66 |
| A_69_P067841 | 1,73E-03 | KIAA1543 | 0,66 |
| Oligo-245 | 8,14E-04 | MRPS7 | 0,66 |
| A_69_P088732 | 4,90E-03 | PALLD | 0,66 |
| Oligo-326 | 1,52E-03 | SLC25A17 | 0,66 |
| A_69_P018566 | 4,37E-05 | ZNF771 | 0,66 |
| A_69_P101001 | 3,62E-03 | CLEC14A | 0,67 |
| A_69_P044811 | 1,83E-03 | GPR20 | 0,67 |
| A_69_P047644 | 1,36E-03 | LIM2 | 0,67 |
| A_69_P086676 | 3,46E-03 | NR0B2 | 0,67 |
| A_69_P110896 | 1,04E-03 | RBBP6 | 0,67 |
| A_69_P049951 | 4,16E-04 | SGK1 | 0,67 |
| A_69_P029336 | 4,55E-03 | TPPP | 0,67 |
| A_69_P064291 | 1,85E-03 | YDJC | 0,67 |
| A_69_P078251 | 3,68E-03 | ZMIZ2 | 0,67 |
| A_69_P013661 | 3,75E-03 | AKNA | 0,68 |
| A_69_P007006 | 2,63E-04 | C19orf20 | 0,68 |
| A_69_P025146 | 3,93E-03 | C20orf118 | 0,68 |
| A_69_P086426 | 4,96E-03 | HCRTR1 | 0,68 |
| A_69_P014946 | 1,25E-03 | KCNT1 | 0,68 |
| A_69_P011071 | 3,28E-03 | LTBP2 | 0,68 |
| A_69_P020131 | 3,61E-03 | MSLNL | 0,68 |
| A_69_P094161 | 2,11E-03 | NANOS1 | 0,68 |
| A_69_P096276 | 6,25E-04 | PCNXL2 | 0,68 |
| A_69_P057635 | 2,15E-03 | PFN2 | 0,68 |
| A_69_P094581 | 1,10E-03 | PITX3 | 0,68 |
| A_69_P055966 | 1,43E-03 | C3orf54 | 0,69 |
| A_69_P087890 | 2,13E-03 | CCDC27 | 0,69 |
| A_69_P034381 | 1,11E-03 | CCL14 | 0,69 |
| A_69_P056661 | 1,78E-03 | CCR8 | 0,69 |
| A_69_P067751 | 3,25E-03 | CRB3 | 0,69 |
| Oligo-79 | 3,19E-03 | CSF1 | 0,69 |
| A_69_P016466 | 3,83E-03 | KRTAP26-1 | 0,69 |
| A_69_P116086 | 5,73E-04 | NPDC1 | 0,69 |
| A_69_P034939 | 4,98E-03 | NXN | 0,69 |
| A_69_P024906 | 2,13E-03 | PXMP4 | 0,69 |
| A_69_P031936 | 5,41E-04 | RECQL5 | 0,69 |
| A_69_P031896 | 4,42E-04 | TRIM47 | 0,69 |
| A_69_P023771 | 1,71E-03 | AKT1 | 0,70 |
| A_69_P003926 | 3,92E-03 | APH1B | 0,70 |
| A_69_P004472 | 4,97E-03 | C17orf64 | 0,70 |
| A_69_P063102 | 3,62E-03 | C5orf13 | 0,70 |
| A_69_P033946 | 1,27E-03 | CA10 | 0,70 |
| A_69_P074766 | 1,10E-04 | CCDC19 | 0,70 |
| A_69_P010376 | 4,16E-03 | CD6 | 0,70 |
| A_69_P084621 | 2,19E-03 | CPZ | 0,70 |
| A_69_P126316 | 4,48E-03 | CRX | 0,70 |
| A_69_P010831 | 1,62E-03 | DNAJC4 | 0,70 |
| A_69_P100951 | 1,88E-03 | INSM2 | 0,70 |
| A_69_P127368 | 1,29E-03 | MT4 | 0,70 |
| A_69_P083616 | 4,43E-03 | STAP1 | 0,70 |
| A_69_P067811 | 9,04E-04 | TRAPPC5 | 0,70 |
| A_69_P111342 | 3,42E-03 | Csnk2a1 | 0,71 |
| A_69_P050281 | 3,20E-03 | CTDSP1 | 0,71 |
| A_69_P046416 | 3,59E-03 | DMRTC2 | 0,71 |
| A_69_P064651 | 3,53E-05 | GATSL3 | 0,71 |
| A_69_P061981 | 3,94E-03 | GPR151 | 0,71 |
| A_69_P000006 | 3,80E-03 | HTR1B | 0,71 |
| A_69_P055532 | 1,89E-03 | LRTM1 | 0,71 |
| A_69_P055731 | 5,43E-04 | PCBP4 | 0,71 |
| A_69_P032416 | 4,74E-03 | SCN4A | 0,71 |
| A_69_P003891 | 2,59E-04 | SMAD6 | 0,71 |
| A_69_P016881 | 4,94E-03 | TFF3 | 0,71 |
| A_69_P011006 | 2,71E-03 | TM7SF2 | 0,71 |
| A_69_P099056 | 3,70E-04 | C15orf52 | 0,72 |
| A_69_P103461 | 3,36E-03 | C16orf72 | 0,72 |
| Oligo-44 | 2,10E-03 | C1QTNF2 | 0,72 |
| A_69_P093756 | 1,86E-03 | CALY | 0,72 |
| A_69_P020151 | 2,97E-03 | CCDC78 | 0,72 |
| A_69_P081556 | 1,39E-03 | CES3 | 0,72 |
| A_69_P106120 | 1,21E-03 | LZTS2 | 0,72 |
| A_69_P059341 | 2,42E-03 | PELI1 | 0,72 |
| A_69_P030911 | 1,86E-03 | PVRL3 | 0,72 |
| A_69_P046341 | 2,19E-03 | TMEM145 | 0,72 |
| A_69_P089846 | 2,53E-05 | ASMT | 0,73 |
| A_69_P006376 | 3,57E-03 | ATP1A3 | 0,73 |
| A_69_P028857 | 9,21E-04 | EGFLAM | 0,73 |
| A_69_P038320 | 2,40E-03 | HLA-DRB1 | 0,73 |
| Oligo-172 | 3,05E-03 | IL4R | 0,73 |
| A_69_P048186 | 1,88E-03 | ISOC2 | 0,73 |
| A_69_P058161 | 3,16E-03 | KIAA1310 | 0,73 |
| A_69_P068037 | 4,24E-03 | MAML2 | 0,73 |
| A_69_P010796 | 4,61E-03 | OTUB1 | 0,73 |
| A_69_P004861 | 8,16E-04 | OXT | 0,73 |
| A_69_P125576 | 3,42E-04 | PRDM16 | 0,73 |
| A_69_P052935 | 3,87E-03 | RHEBL1 | 0,73 |
| A_69_P015271 | 2,36E-03 | SLC20A2 | 0,73 |
| A_69_P110168 | 2,81E-03 | SMYD3 | 0,73 |
| A_69_P021191 | 9,78E-04 | TST | 0,73 |
| A_69_P037756 | 5,77E-04 | ZFP57 | 0,73 |
| A_69_P123876 | 2,95E-03 | C7orf47 | 0,74 |
| A_69_P086377 | 9,17E-05 | CCDC28B | 0,74 |
| A_69_P021601 | 7,69E-04 | CSDC2 | 0,74 |
| Oligo-82 | 4,99E-03 | CSK | 0,74 |
| A_69_P114886 | 2,12E-03 | PGS1 | 0,74 |
| A_69_P056316 | 1,58E-03 | PRSS50 | 0,74 |
| A_69_P084546 | 2,55E-03 | S100P | 0,74 |
| A_69_P055915 | 3,24E-03 | SEMA3F | 0,74 |
| A_69_P056731 | 2,03E-03 | SLC22A13 | 0,74 |
| A_69_P024961 | 4,52E-03 | TP53INP2 | 0,74 |
| A_69_P095946 | 1,83E-03 | ZMIZ1 | 0,74 |
| A_69_P054361 | 1,61E-04 | MARCH9 | 0,75 |
| A_69_P030681 | 1,99E-03 | ADPRH | 0,75 |
| A_69_P075446 | 4,40E-03 | CHRNB2 | 0,75 |
| A_69_P086366 | 7,95E-04 | DCDC2B | 0,75 |
| A_69_P010641 | 1,37E-03 | GNG3 | 0,75 |
| A_69_P102571 | 2,03E-03 | HOOK2 | 0,75 |
| A_69_P074741 | 3,03E-03 | KCNJ10 | 0,75 |
| A_69_P022161 | 1,31E-03 | LGALS3 | 0,75 |
| A_69_P020106 | 3,09E-03 | LMF1 | 0,75 |
| A_69_P071051 | 2,56E-03 | LRRC32 | 0,75 |
| A_69_P000742 | 2,47E-03 | LY49F | 0,75 |
| A_69_P087341 | 4,16E-03 | RSG1 | 0,75 |
| A_69_P044632 | 2,12E-04 | SQLE | 0,75 |
| A_69_P028141 | 2,22E-03 | TMEM59L | 0,75 |
| A_69_P053176 | 2,85E-03 | ACVR1B | 0,76 |
| A_69_P052881 | 1,87E-03 | CACNB3 | 0,76 |
| A_69_P086966 | 4,42E-04 | LYPLA2 | 0,76 |
| A_69_P086331 | 3,19E-04 | MARCKSL1 | 0,76 |
| A_69_P102906 | 1,81E-03 | PTOV1 | 0,76 |
| A_69_P004696 | 1,14E-03 | SMAD9 | 0,76 |
| A_69_P129476 | 3,89E-04 | TPM1 | 0,76 |
| A_69_P038476 | 4,48E-03 | BAK1 | 0,77 |
| A_69_P036361 | 1,79E-03 | C17orf103 | 0,77 |
| A_69_P045636 | 3,19E-03 | CLIP3 | 0,77 |
| A_69_P000841 | 7,88E-04 | COMT | 0,77 |
| A_69_P094571 | 1,25E-03 | FBXL15 | 0,77 |
| A_69_P105476 | 7,39E-04 | MPZL1 | 0,77 |
| A_69_P046351 | 4,80E-03 | PAFAH1B3 | 0,77 |
| A_69_P038364 | 3,89E-03 | PSMB9 | 0,77 |
| A_69_P117197 | 3,34E-03 | RAVER1 | 0,77 |
| A_69_P035501 | 1,36E-03 | RNF167 | 0,77 |
| A_69_P051546 | 2,56E-04 | TAPBPL | 0,77 |
| Oligo-344 | 2,09E-03 | TGFB1 | 0,77 |
| A_69_P105409 | 4,80E-03 | Zc3h4 | 0,77 |
| A_69_P008971 | 4,72E-03 | ABTB2 | 0,78 |
| A_69_P010966 | 4,28E-03 | ARL2 | 0,78 |
| A_69_P121836 | 2,19E-03 | ATP6V1G2 | 0,78 |
| A_69_P085566 | 5,45E-04 | B4GALT2 | 0,78 |
| A_69_P025486 | 3,48E-03 | DBNDD2 | 0,78 |
| A_69_P076181 | 9,91E-04 | GJA5 | 0,78 |
| A_69_P037831 | 1,00E-04 | GNL1 | 0,78 |
| A_69_P129656 | 4,75E-03 | GPX1 | 0,78 |
| Oligo-187 | 3,83E-03 | LCK | 0,78 |
| A_69_P038056 | 2,33E-03 | LTB | 0,78 |
| A_69_P045146 | 4,46E-03 | MFSD3 | 0,78 |
| A_69_P018696 | 4,01E-04 | PPP4C | 0,78 |
| A_69_P018596 | 3,19E-03 | QPRT | 0,78 |
| A_69_P118516 | 4,05E-03 | SPNS2 | 0,78 |
| A_69_P097892 | 4,69E-03 | TMEM202 | 0,78 |
| A_69_P015137 | 1,71E-03 | C9orf167 | 0,79 |
| A_69_P025881 | 3,47E-03 | CBLN4 | 0,79 |
| A_69_P031356 | 2,03E-03 | CD7 | 0,79 |
| A_69_P046241 | 4,12E-03 | CYP2S1 | 0,79 |
| A_69_P018171 | 4,28E-03 | ELN | 0,79 |
| A_69_P051271 | 8,27E-04 | FBXL14 | 0,79 |
| A_69_P055868 | 2,15E-03 | HYAL2 | 0,79 |
| A_69_P054233 | 1,41E-03 | NAB2 | 0,79 |
| A_69_P065476 | 1,94E-03 | ORAI1 | 0,79 |
| A_69_P027986 | 4,84E-03 | PGLS | 0,79 |
| Oligo-309 | 3,69E-03 | PYGL | 0,79 |
| A_69_P008011 | 2,48E-03 | STX11 | 0,79 |
| A_69_P074761 | 7,54E-04 | TAGLN2 | 0,79 |
| A_69_P031741 | 4,31E-03 | TMC6 | 0,79 |
| A_69_P010667 | 2,35E-03 | TMEM179B | 0,79 |
| Oligo-364 | 3,96E-03 | TP53 | 0,79 |
| A_69_P092636 | 3,29E-03 | ANKRD58 | 0,80 |
| A_69_P006826 | 2,18E-03 | C19orf22 | 0,80 |
| A_69_P024371 | 2,09E-03 | CENPB | 0,80 |
| A_69_P020386 | 5,46E-04 | GLIPR1 | 0,80 |
| A_69_P061131 | 5,15E-04 | GRK6 | 0,80 |
| A_69_P068611 | 4,36E-03 | IL10RA | 0,80 |
| A_69_P045156 | 1,40E-03 | LRRC14 | 0,80 |
| A_69_P024956 | 3,18E-03 | MAP1LC3A | 0,80 |
| A_69_P018716 | 1,87E-04 | MAPK3 | 0,80 |
| A_69_P106401 | 1,40E-03 | NFIX | 0,80 |
| A_69_P010826 | 1,71E-03 | NUDT22 | 0,80 |
| A_69_P011381 | 1,94E-03 | RPS6KB2 | 0,80 |
| A_69_P055011 | 2,06E-03 | RPUSD3 | 0,80 |
| A_69_P010946 | 4,07E-03 | SAC3D1 | 0,80 |
| A_69_P035091 | 2,99E-03 | SRR | 0,80 |
| A_69_P034781 | 2,33E-03 | TRAF4 | 0,80 |
| A_69_P011626 | 3,98E-04 | TSPAN32 | 0,80 |
| A_69_P032061 | 4,54E-03 | USH1G | 0,80 |
| A_69_P096911 | 2,26E-04 | BLM | 1,20 |
| A_69_P015331 | 2,19E-03 | GINS4 | 1,20 |
| A_69_P099866 | 4,38E-03 | RAB2B | 1,20 |
| A_69_P061192 | 1,15E-04 | UIMC1 | 1,20 |
| A_69_P076258 | 3,97E-03 | WARS2 | 1,20 |
| A_69_P004985 | 2,62E-03 | HSPA8 | 1,21 |
| A_69_P012271 | 3,49E-03 | NSUN6 | 1,21 |
| A_69_P012687 | 8,17E-04 | PITRM1 | 1,21 |
| A_69_P101026 | 1,91E-03 | PNN | 1,21 |
| A_69_P083851 | 1,27E-04 | SLAIN2 | 1,21 |
| A_69_P103071 | 1,54E-03 | CCND3 | 1,22 |
| A_69_P107357 | 1,23E-04 | DDX18 | 1,22 |
| A_69_P079571 | 7,53E-04 | ING3 | 1,22 |
| A_69_P020771 | 3,70E-04 | NEDD1 | 1,22 |
| A_69_P093936 | 1,49E-03 | FAM175B | 1,23 |
| A_69_P076576 | 4,55E-03 | LRIF1 | 1,23 |
| A_69_P087662 | 1,36E-03 | NMNAT1 | 1,23 |
| A_69_P059946 | 4,32E-03 | FEZ2 | 1,24 |
| A_69_P078307 | 3,23E-03 | HUS1 | 1,24 |
| A_69_P092956 | 4,43E-03 | MBNL3 | 1,24 |
| A_69_P040378 | 1,01E-03 | NUDT15 | 1,24 |
| A_69_P105117 | 2,80E-03 | SLAMF7 | 1,24 |
| A_69_P082871 | 2,96E-03 | SMARCAD1 | 1,24 |
| A_69_P042196 | 1,14E-03 | TLK1 | 1,24 |
| A_69_P058106 | 4,54E-03 | TMEM131 | 1,24 |
| A_69_P003732 | 2,45E-03 | GOT1 | 1,25 |
| A_69_P048631 | 1,97E-03 | MTO1 | 1,25 |
| A_69_P007696 | 3,06E-03 | PDCD2 | 1,25 |
| A_69_P015508 | 9,47E-04 | ZFP36L2 | 1,25 |
| A_69_P036846 | 7,41E-04 | CDKAL1 | 1,26 |
| A_69_P034363 | 1,03E-05 | HEATR6 | 1,26 |
| A_69_P032341 | 4,92E-03 | HELZ | 1,26 |
| A_69_P113502 | 3,59E-03 | LINC00152 | 1,26 |
| A_69_P036231 | 1,87E-03 | SHMT1 | 1,26 |
| A_69_P095787 | 5,01E-04 | DNAJC9 | 1,27 |
| A_69_P065616 | 1,67E-03 | MPHOSPH9 | 1,27 |
| A_69_P040066 | 3,75E-03 | RFC3 | 1,27 |
| A_69_P086161 | 5,90E-06 | SFPQ | 1,27 |
| A_69_P057241 | 1,43E-03 | TOPBP1 | 1,27 |
| A_69_P072851 | 4,39E-03 | UEVLD | 1,27 |
| A_69_P063686 | 8,41E-05 | GFM2 | 1,28 |
| A_69_P095051 | 3,56E-04 | HELLS | 1,28 |
| A_69_P101861 | 2,30E-03 | ITM2C | 1,28 |
| A_69_P063477 | 1,48E-03 | MSH3 | 1,28 |
| A_69_P071421 | 2,33E-03 | RRM1 | 1,28 |
| A_69_P128771 | 3,52E-03 | AKAP9 | 1,29 |
| A_69_P040001 | 2,97E-03 | HSPH1 | 1,29 |
| A_69_P033466 | 4,33E-03 | MED1 | 1,29 |
| A_69_P070936 | 9,85E-05 | PRCP | 1,29 |
| A_69_P008586 | 4,24E-03 | TROVE2 | 1,29 |
| A_69_P022151 | 1,18E-03 | WDHD1 | 1,29 |
| A_69_P120636 | 2,96E-03 | FAM126B | 1,30 |
| A_69_P018931 | 2,87E-03 | PALB2 | 1,30 |
| A_69_P013236 | 2,08E-03 | RNF20 | 1,30 |
| A_69_P024181 | 1,84E-03 | ESF1 | 1,31 |
| A_69_P059966 | 1,51E-03 | FAM98A | 1,31 |
| A_69_P016416 | 3,53E-03 | LTN1 | 1,31 |
| A_69_P058957 | 3,11E-04 | MOBKL1B | 1,31 |
| A_69_P091326 | 1,14E-03 | PJA1 | 1,31 |
| A_69_P086601 | 4,93E-03 | FAM76A | 1,32 |
| A_69_P077236 | 4,62E-03 | HS2ST1 | 1,32 |
| A_69_P074221 | 3,31E-04 | INTS7 | 1,32 |
| Oligo-281 | 8,64E-04 | PEA15 | 1,32 |
| A_69_P028497 | 3,92E-03 | ELOVL7 | 1,33 |
| A_69_P024312 | 1,72E-03 | PCNA | 1,33 |
| A_69_P078961 | 7,97E-04 | SNX13 | 1,33 |
| A_69_P035436 | 8,29E-05 | C1QBP | 1,34 |
| A_69_P036757 | 1,75E-03 | MYLIP | 1,34 |
| A_69_P022596 | 4,14E-04 | RAD51B | 1,34 |
| A_69_P054637 | 4,18E-03 | SEC61A1 | 1,34 |
| A_69_P093071 | 2,42E-03 | DDX26B | 1,35 |
| A_69_P109611 | 2,92E-03 | BHLHE41 | 1,36 |
| A_69_P054476 | 3,11E-03 | GNS | 1,36 |
| A_69_P058306 | 1,51E-03 | NPHP1 | 1,36 |
| A_69_P042896 | 1,69E-03 | ORC2 | 1,36 |
| A_69_P058661 | 2,45E-04 | PTCD3 | 1,36 |
| A_69_P049241 | 3,68E-03 | LACE1 | 1,37 |
| A_69_P112859 | 4,41E-03 | PIKFYVE | 1,37 |
| A_69_P096028 | 1,88E-04 | C1orf96 | 1,38 |
| A_69_P084416 | 2,70E-03 | HS3ST1 | 1,38 |
| A_69_P063776 | 3,91E-03 | MCCC2 | 1,38 |
| A_69_P024282 | 6,52E-04 | MCM8 | 1,38 |
| A_69_P095737 | 3,94E-03 | DDIT4 | 1,39 |
| A_69_P059206 | 4,33E-05 | GFPT1 | 1,39 |
| A_69_P038631 | 2,93E-03 | MAPK14 | 1,39 |
| A_69_P107546 | 7,80E-04 | NRP2 | 1,39 |
| A_69_P074202 | 6,88E-04 | TMEM206 | 1,39 |
| A_69_P083046 | 4,67E-03 | ARHGAP24 | 1,40 |
| A_69_P083967 | 9,82E-04 | ATP8A1 | 1,40 |
| A_69_P065856 | 1,89E-03 | GOLGA3 | 1,41 |
| A_69_P092296 | 2,95E-03 | RBM41 | 1,41 |
| A_69_P000926 | 1,35E-03 | PECAM1 | 1,42 |
| A_69_P089041 | 3,62E-04 | PET112 | 1,42 |
| A_69_P072894 | 3,91E-03 | E2F8 | 1,43 |
| A_69_P084301 | 4,49E-03 | NCAPG | 1,43 |
| A_69_P032988 | 6,53E-04 | PSMC3IP | 1,43 |
| A_69_P110487 | 3,14E-03 | Zbtb10 | 1,43 |
| A_69_P087007 | 2,24E-03 | ZNF436 | 1,43 |
| A_69_P089476 | 1,09E-03 | KIAA1109 | 1,44 |
| A_69_P054481 | 3,42E-03 | TBC1D30 | 1,44 |
| A_69_P116114 | 3,18E-04 | C18orf25 | 1,45 |
| A_69_P044201 | 4,60E-04 | RGS22 | 1,45 |
| A_69_P042851 | 8,19E-04 | SGOL2 | 1,45 |
| A_69_P040417 | 6,08E-04 | ZC3H13 | 1,45 |
| A_69_P122867 | 9,68E-04 | ACYP1 | 1,46 |
| A_69_P008176 | 4,29E-03 | EXO1 | 1,47 |
| A_69_P010496 | 3,56E-03 | FEN1 | 1,47 |
| A_69_P059652 | 9,41E-04 | MCFD2 | 1,47 |
| A_69_P110316 | 4,89E-03 | AHNAK | 1,48 |
| A_69_P096467 | 7,84E-04 | ERCC6 | 1,48 |
| A_69_P082707 | 1,29E-03 | CENPE | 1,49 |
| A_69_P019022 | 2,18E-03 | ERI2 | 1,50 |
| A_69_P025001 | 1,76E-03 | PROCR | 1,50 |
| A_69_P043717 | 3,39E-03 | UBXN2B | 1,50 |
| A_69_P111736 | 3,55E-03 | KIAA0753 | 1,51 |
| A_69_P059761 | 2,87E-03 | THADA | 1,51 |
| A_69_P039761 | 8,83E-04 | SKA3 | 1,52 |
| A_69_P074421 | 3,83E-03 | NUF2 | 1,53 |
| A_69_P028502 | 3,05E-03 | DEPDC1B | 1,55 |
| A_69_P068232 | 1,02E-03 | RAB39 | 1,55 |
| A_69_P024717 | 6,42E-04 | TPX2 | 1,55 |
| A_69_P129341 | 3,72E-03 | FCRL3 | 1,56 |
| A_69_P113095 | 1,54E-04 | KIAA1671 | 1,56 |
| A_69_P079591 | 2,16E-03 | FAM3C | 1,57 |
| A_69_P015856 | 2,33E-03 | KLKB1 | 1,57 |
| A_69_P056886 | 7,01E-04 | OSBPL10 | 1,58 |
| A_69_P060397 | 1,75E-03 | CENPO | 1,59 |
| A_69_P122131 | 8,14E-04 | DDX60L | 1,59 |
| A_69_P013262 | 8,05E-04 | SMC2 | 1,59 |
| A_69_P054971 | 3,90E-03 | FANCD2 | 1,60 |
| A_69_P000123 | 4,79E-03 | CGA | 1,61 |
| A_69_P055426 | 3,28E-03 | FLNB | 1,61 |
| A_69_P018391 | 4,47E-03 | ITGAD | 1,61 |
| A_69_P066111 | 1,87E-03 | NPC1 | 1,61 |
| A_69_P085386 | 1,76E-03 | TSPAN1 | 1,62 |
| A_69_P061679 | 4,64E-05 | HAVCR1 | 1,65 |
| A_69_P033342 | 8,22E-04 | CDC6 | 1,68 |
| A_69_P032963 | 3,07E-03 | CCR10 | 1,69 |
| A_69_P089011 | 4,90E-03 | TRIM2 | 1,69 |
| A_69_P080996 | 1,13E-03 | SHCBP1 | 1,74 |
| A_69_P013427 | 1,20E-04 | CTNNAL1 | 1,75 |
| A_69_P008657 | 4,61E-03 | ASPM | 1,83 |
| A_69_P026625 | 3,00E-03 | TLE1 | 1,86 |
| A_69_P012446 | 7,98E-04 | MCM10 | 1,88 |
| A_69_P026876 | 7,89E-05 | VLDLR | 1,91 |
| A_69_P008332 | 2,37E-04 | ENAH | 1,96 |
| A_69_P080056 | 3,03E-04 | ATP6V0A4 | 1,97 |
| A_69_P016829 | 3,90E-03 | BACE2 | 2,03 |
| A_69_P029546 | 1,00E-05 | SERPINI1 | 2,05 |
| A_69_P021653 | 2,39E-03 | SEPT3 | 2,17 |
| A_69_P003546 | 3,01E-03 | IFI6 | 2,17 |
| A_69_P056687 | 4,38E-03 | TTC21A | 2,31 |
| A_69_P042836 | 3,04E-03 | SPATS2L | 2,44 |
| A_69_P011706 | 4,12E-03 | IRF7 | 2,63 |
| A_69_P051172 | 1,54E-03 | USP18 | 2,92 |
