# Supplementary material for: Behavioral and Transcriptomic Fingerprints of an Enriched Environment in Horses (Equus caballus)
Source: PLoS One. 2014 Dec 10;9(12):e114384. doi: 10.1371/journal.pone.0114384 (PMC4262392; doi:10.1371/journal.pone.0114384)
Supplement: Table S3 — IPA core analysis, direct and indirect: Enriched (EE-treated) group. (DOCX) [file pone.0114384.s003.docx]

**Table S3:** IPA core analysis, direct and indirect: Enriched (EE-treated) group

**Top Networks**

***Associated Network Functions* *Score***

1 Cell Signaling, Post-Translational Modification, Cellular Development 36

2 Cancer, Cellular Growth and Proliferation, Immunological Disease 34

3 Infectious Disease, Respiratory Disease, Embryonic Development 26

4 Cellular Assembly and Organization, Cellular Function and Maintenance, Cellular Compromise 25

**Molecular and Cellular Functions**

***Name p-value # Molecules***

Cellular Growth and Proliferation 2,20E-05 - 1,02E-02 81

Cellular Movement 2,30E-05 - 1,03E-02 42

Cell-To-Cell Signaling and Interaction 3,32E-05 - 9,52E-03 49

Cell Signaling 4,50E-05 - 9,75E-03 31

Molecular Transport 4,50E-05 - 9,75E-03 58

**Physiological System Development and Function**

***Name p-value # Molecules***

Tissue Morphology 6,55E-05 - 9,62E-03 66

Skeletal and Muscular System Development and Function 7,97E-05 - 9,57E-03 37

Behavior 1,21E-04 - 8,11E-03 24

Nervous System Development and Function 1,21E-04 - 9,57E-03 40

Hematological System Development and Function 1,29E-04 - 1,03E-02 55

Summary**Top Canonical Pathways**

***Name p-value Ratio***

TGF-β Signaling 3,97E-04 6/89 (0,067)

Factors Promoting Cardiogenesis in Vertebrates 5,43E-04 6/95 (0,063)

Molecular Mechanisms of Cancer 6,26E-04 12/381 (0,031)

Wnt/b-catenin Signaling 8,48E-04 8/175 (0,046)

Role of Osteoblasts, Osteoclasts

and Chondrocytes in Rheumatoid Arthritis 1,03E-03 9/240 (0,038)
