# Supplementary material for: Behavioral and Transcriptomic Fingerprints of an Enriched Environment in Horses (Equus caballus)
Source: PLoS One. 2014 Dec 10;9(12):e114384. doi: 10.1371/journal.pone.0114384 (PMC4262392; doi:10.1371/journal.pone.0114384)
Supplement: Table S4 — IPA core analysis, direct and indirect: Control group. (DOCX) [file pone.0114384.s004.docx]

**Table S4:** IPA core analysis, direct and indirect: Control group

**Top Networks**

***Associated Network Functions* *Score***

1 Cell Cycle, DNA Replication, Recombination, and Repair, Cellular Response to Therapeutics 58

2 Embryonic Development, Organ Development, Organ Morphology 41

3 Cellular Assembly and Organization, Cellular Function and Maintenance, Developmental Disorder 36

4 Cell Cycle, Hepatic System Development and Function, Nucleic Acid Metabolism 28

**Molecular and Cellular Functions**

***Name p-value # Molecules***

DNA Replication, Recombination, and Repair 2,34E-06 - 4,62E-02 35

Cell Cycle 2,86E-06 - 4,80E-02 43

Cellular Assembly and Organization 1,10E-05 - 4,02E-02 22

**Physiological System Development and Function**

***Name p-value # Molecules***

Embryonic Development 5,45E-05 - 4,64E-02 30

Organismal Survival 5,45E-05 - 5,52E-03 37

Tissue Development 6,62E-05 - 4,02E-02 19

**Top Canonical Pathways**

***Name p-value Ratio***

Mismatch Repair in Eukaryotes 1,37E-07 5/24 (0,208)

ATM Signaling 1,29E-04 5/62 (0,081)

Role of CHK Proteins in Cell Cycle,Checkpoint Control 1,05E-03 4/57 (0,07)

Role of BRCA1in DNA Damage Response 1,46E-03 4/65 (0,062)

p53 Signaling 7,09E-03 4/96 (0,042)
