# Supplementary material for: Behavioral and Transcriptomic Fingerprints of an Enriched Environment in Horses (Equus caballus)
Source: PLoS One. 2014 Dec 10;9(12):e114384. doi: 10.1371/journal.pone.0114384 (PMC4262392; doi:10.1371/journal.pone.0114384)
Supplement: File S1 — Supplementary methods. (DOCX) [file pone.0114384.s005.docx]

**Supplementary methods**

**Animals and experimental groups**

Before the start of the experiment, the 19 foals were reared together, kept outdoors all summer and indoors during winter at the experimental INRA Unit in Nouzilly (France). When stabled during winter, they all received concentrated pellets and hay, and were turned out to a paddock 7 hours per day. They were weaned at 7 months of age. Prior to the experiment, animals were subjected to exactly the same routine management (fed every day and haltered about once a month). The horses were then randomly allocated to either the EE-treated group (*n* = 10, four males and six females) or the control group (*n* = 9, six males and three females). In order to compile groups balanced for personality, the week before the beginning of the treatment, the horses were subjected to a series of preliminary behavioral tests during three consecutive days (Fig. 1). These tests measured reactivity to a human (passive and active human tests), sensory sensitivity (tactile sensitivity tests), fearfulness (novel object and surprise tests), gregariousness (social isolation test), and locomotor activity (evaluated by the distance covered during all the tests), see ([1](#_ENREF_1)) for the description of the tests.

Behavioral observations in the home box

During the first five weeks of experimentation (Figure 1), a horse’s behavioral patterns were observed by scan-sampling from Monday to Friday. The scans were performed for 90 min per day, from 0900 h to 1030 h, 1030 h to 1200 h, 1200 h to 1330 h, 1330 h to 1500 h, or 1500 h to 1630 h (new successive time slot each day). All observations were made by a single observer. Each horse was observed every 5 minutes, and we observed the presence or absence of the following behavioral patterns: vocalizations (neighs), alert postures (elevated neck and ears pointed forward), lying down (ventral or lateral recumbence), ears pointed backward, and aberrant behavior (sniffing/licking box walls or bars, repetitive pawing, kicking the wall, and repetitive head swinging). The occurrences of each of these patterns were summed up for each week. In addition, at the end of weeks 1, 5, and 12, we noted the number of horses per group that did not eat their meal entirely and systematically within the hour after the meal distribution.

**Personality tests**

At the end of weeks 5, 12, and 23, personality tests were performed. The tested horse was led into an 8.10 m × 2.70 m unfamiliar test arena located in a barn adjacent to the stable. Two observers, always the same, were hidden behind a dark window, and an audience horse was tied up outside the box, visible to the tested horse. We analyzed the behavioral parameters selected during these studies, as they appear to be reliable indicators of a horse’s personality because of their stability across time and situations ([2-5](#_ENREF_2)). After a familiarization phase during which the horse was free in the box for 360 s, the tests occurred exactly in the order presented below.

**Passive and active human test**. To characterize reactivity to humans ([2](#_ENREF_2)), an unknown experimenter (always the same person) entered the arena and remained motionless beside a wall for 180 s. The number of contacts with the human (sniffing or nibbling the experimenter) was recorded. Then, he moved slowly towards the horse and tried to put a halter. The time taken to put the halter was recorded.

**Von Frey test filament assay.** To characterize tactile sensitivity ([5](#_ENREF_5)), an experimenter applied a von Frey ﬁlament to the base of the horse’s withers, while the horse was held on a lunge by a second experimenter (von Frey ﬁlaments, Stoelting, IL, USA). These ﬁlaments consist of a hard plastic body connected to a nylon thread. The purpose of this test is to evaluate the response to mechanical stimuli using filaments of varying strengths. Thus, the filaments are calibrated to exert specific magnitudes of force on the skin, ranging from 0.008 to 300 g. They were applied perpendicularly to the animal’s skin until the nylon filament started to bend, and then was withdrawn. The trembling of the platysma muscle was encoded as follows (0: not trembling; 0.5: trembling only when the filament was withdrawn; 1: trembling when the filament was applied and withdrawn). The test had two phases: In the first phase, which was carried out after the passive human test, a 0.008-g filament was applied to the right side of the horse, followed by application of a 300-g filament to the left side. In the second phase, after the novel area test, a 0.02-g filament was applied to the horse’s right side, followed by a 1-g filament to the left side. The responses to the 4 filaments were recorded.

**The stifle-haunch axis stimulation test**. To characterize tactile sensitivity ([5](#_ENREF_5)), we performed a test consisting of stimulating the stifle-haunch axis with four different 3-cm wide instruments composed of different material (brush, plastic, rubber, cotton). The horse was held on a lunge during the test. The experimenter quickly moved the instrument upwards along the tuft of the stifle-haunch axis, keeping a slight but even pressure. The intensity of the bodily reaction was assessed on a predefined scale; see ([5](#_ENREF_5)) for more details.

**The novel object test**. To characterize fearfulness ([3](#_ENREF_3)), a novel object (week 5: colored plastic pieces attached to a 1-m–long bar; week 12: green and yellow carton cube (0.8 m × 1.2 m × 0.4 m); week 23: a brilliant shiny tinsel attached to a 1-m-long wooden tube) was placed near the entrance to the arena for 180 s. The numbers of contacts (sniffing or nibbling the object) and glances at the object were recorded.

**The social isolation test.** To characterize gregariousness ([4](#_ENREF_4)), the audience horse was led by an experimenter outside to the barn so that it would become invisible and inaudible to the tested horse for 90 s. The number of vocalizations (neighs) was recorded.

**The novel area test**. For this test, which evaluates fearfulness ([3](#_ENREF_3)), the ﬂoor of the novel arena was divided into three zones of 2.7 m × 2.7 m. The first through the third zones were the start, intermediate, and arrival zones respectively. The arrival zone contained a bucket of the pellets with which the horses were familiar. Immediately prior to the test, the horses underwent a habituation phase during which they learned how to go from the start zone to the arrival zone containing the bucket. To achieve this result, an experimenter led the horse by halter to the start zone and released it so that it was free to go to the arrival zone to eat. This action was repeated three times. During the test, a pink carpet (2 m × 2.7 m) was placed in the intermediate zone. As in the habituation phase, the experimenter released the horse in the start zone. The time until the horse ate from the bucket was recorded. If the horse did not eat within 180 s, the test was terminated and a time of 180 s was assigned.

**The suddenness test.** In this test, which evaluates fearfulness ([3](#_ENREF_3)), a black umbrella was suddenly opened in front of the animal while it was eating. A bucket of pellets was placed near the arena’s entrance. After the animal had been eating with its head in the bucket for 3 s, the experimenter, who was not visible to the horse, opened the umbrella. We recorded the latency to eat again from the bucket after the umbrella opening.

**Locomotor activity**. In order to measure locomotor activity, we divided the test pen into six sectors of equal size. We recorded the number of sectors crossed by one of the horse’s front hooves during the novel arena test, the novel object test, and the passive human test.

**The handling test.** At the end of week 5, a test consisting of fitting the horse with a halter and leading him/her to a paddock was performed alone (on a separate day). The number of defensive reactions (startle responses, rearing, sudden veering, bites, kicks, head kicks, and tentative to escape) was recorded.

**Learning**

**The learning apparatus.** The tested horse was individually maintained with two leads (1.2 m long) in a familiar box (2.7 m × 8.1 m) in front of an open door blocked by a wooden plank 1.2 m high. Two experimenters (A and B), a man and a woman, sat down in front of the horse and conducted the trials alternately. Two orange traffic cones (0.45 m high) separated by 0.4 m were placed on a horizontal wooden plank fixed at a height of 0.3 m above the ground. These traffic cones were placed between the horse and the experimenters such that the horse could touch the cones but not the experimenters. The experimenter’s side of the man and the woman was randomly allocated to each horse and was maintained for the whole learning procedure.

**The familiarization phase.** Prior to learning, the horses were familiarized with the apparatus during 10-min sessions. Once per session, each experimenter randomly offered the horse to eat pellets from his hand and the latency to eat the pellets was recorded. All the horses were submitted to a minimum of 5 familiarization sessions on consecutive days or until they accepted the offer to eat the pellets in less than 20 s from the hand of each experimenter. The horse was subjected to the first learning session the next day.

**The learning procedure.** Horses were submitted to exactly 10 learning sessions regularly distributed over 6 weeks. Each session was composed of 30 trials, 15 for each experimenter. The learning procedure consisted of subjecting the horses to a series of two consecutive stages.

The shaping phase (A+, B+). Both experimenters A and B reinforced the animal’s response (touch the suggested cone) with a food reward. At the beginning of each trial, one of the two experimenters shook a bucket of pellets placed under his chair in order to attract the attention of the horse. The trial started as soon as the horse looked at him or after a maximum of three shakes. He then took food from his pocket and pointed to one of the two cones (the target cone) with gestures, until the horse touched the target cone with its nose. First, he moved his forefinger up and down in the direction of the cone (7 times in 10 s), then he repeated the action with his forearm the same number of times, and then with his entire arm. If the horse did not touch the target cone, he tapped the top of the cone with his finger the same number of times. Finally, if the horse did not touch the cone after these gestural instructions, the experimenter tried to attract it to the cone with the food either until the horse touched the target cone or for a maximum of 30 s. The trial ended as soon as the horse touched the target cone with its nose or after the end of all attempted instructions. When the horse touched the target cone, it received a handful of pellets. A trial was assumed to be successful when the horse touched the target cone after the gestural instructions without the use of food, otherwise the trial was labeled as an absence of response. Two trials were spaced at 10 s. The shaping phase criterion consisted of six successful trials out of seven consecutive trials (three successful trials with each experimenter A+ and B+). As soon as the horse reached a step criterion, it was immediately subjected to the next step from the following trial.

The Go/no-go phase (A+, B−). Experimenter A reinforced the animal’s response with a food reward (A+), whereas experimenter B never reinforced (B−). Otherwise, the procedure of this trial was the same as in the simple step, except that the experimenter never attracted the horse with food. The Go-no-go criterion was to perform three successful trials with experimenter A+ and to show the absence of response in three trials with experimenter B−, out of seven consecutives trials. Even when a horse fulfilled the criterion, it continued to be tested until the end of the sessions.

The trial order and the side of the targeted cone were randomly assigned for each session to have no more than two consecutive trials with the same experimenter and/or with the same cone. We recorded the number of horses in the two groups that fulfilled each of the criteria and the proportion of successful (i.e. when the horse touched the target cone after the gestural instructions) with each experimenter for each step.

**Cortisol measurement**

The samples were collected with Salivette® Cortisol (SARSTEDT France). Cotton buds were centrifuged at 3000 g for 20 min at 4°C and the saliva was stored at -20 °C until analysis. Saliva was collected and cortisol was measured in 20 µl samples by using a luminescence immunoassay kit (LIA, IBL, Hamburg, Germany). The measurements were performed with the mean of two replicates. The intra-assay coefficients of variation were 4.8 % and 4.1 % at 1.8 ng / ml and 9.7 ng / ml, respectively. The assay sensitivity was 0.25 ng / ml.

**Statistical and bioinformatic analyses**

**Transcriptomic data**

Because the sequencing of the horse genome was recently completed ([6](#_ENREF_6)), we were able to annotate the microarray. The microarray gene annotations were reanalyzed with Sigreannot-mart, a query environment for expression microarray probe reannotation ([7](#_ENREF_7)). We obtained the annotation of 72% of the probes representing 14,944 unique genes.

In order to identify upstream transcription control pathways mediating observed changes in gene expression, we used two bioinformatic software packages: Transcription Element Listening System (TELiS, <http://telis.ucla.edu>) ([8](#_ENREF_8)) and oPOSSUM (<http://opossum.cisreg.ca/oPOSSUM3/>) ([9](#_ENREF_9), [10](#_ENREF_10)).

The TELiS database contains information on the prevalence of transcription factor-binding motifs (TFBMs) in the promoters of genes from humans and mice. TFBMs are defined by 108 position-specific weight matrices from the JASPAR 2 database or 192 matrices in the TRANSFAC database. The analysis performed was the Transcriptional Shift Analysis in which TFBMs are tested for differential prevalence in the promoters of genes upregulated in the control vs. EE-treated groups (thereby ensuring that all genes analyzed are expressed under at least some conditions in the horse blood cells transcriptome, and thereby mitigating cell type bias with respect to the total population of horse genes, which can otherwise lead to a biased reference group). The results presented were sorted according to statistical significance in a test of differential representation ratios computed over nine combinations of promoter length (−300 bp from transcription start site, −600, and −1000 to +200) and scan stringency (matrix similarity values greater than 0.85, 0.90, and 0.95). A synthetic ratio (geometric average of the various parametric combinations) was used for the graphic representation of the results.

The oPOSSUM single-site analysis tool allows a researcher to identify over-representation of TFBMs and TFBM families within a set of co-expressed genes or sequences generated using high-throughput methods, compared to a background set. The 116 matrices included in oPOSSUM-3 were obtained from the 2010 release of JASPAR database and were constructed from an initial set of 14,083 human–mouse orthologs. The 14,944 unique genes of the microarray constitute the background set. We compared upregulated genes of the EE-treated and the control group to the background set. To detect common TFBMs, we examined 10,000 base pairs upstream and downstream of each transcription start site and a matrix score threshold of 85%. Overrepresented transcription factors were considered significant at a z-score ≥10 and a Fisher score ≤0.01 as suggested by the authors of the software.

To gain further insight into the functional characteristics of the differentially expressed genes, we used the Ingenuity Pathway Analysis software (<http://www.Ingenuity.com>). Ingenuity Pathway Analysis explores interaction-based relationships between a set of genes and produces gene networks. Each network has an associated score derived from a *P* value, indicating the expected likelihood of the genes being present in a network compared to that expected by chance. Scores of two or above have at least 99% likelihood of not being generated by chance.

**References**

1. Lansade L, Simon F (2010) Horses' learning performances are under the influence of several temperamental dimensions. *Appl. Anim. Behav. Sci.* 125(1-2):30-37.

2. Lansade L, Bouissou M-F (2008) Reactivity to humans: A temperament trait of horses which is stable across time and situations. *Appl. Anim. Behav. Sci.* 114(3-4):492-508.

3. Lansade L, Bouissou M-F, Erhard HW (2008) Fearfulness in horses: A temperament trait stable across time and situations. *Appl. Anim. Behav. Sci.* 115(3-4):182-200.

4. Lansade L, Bouissou M-F, Erhard HW (2008) Reactivity to isolation and association with conspecifics: A temperament trait stable across time and situations. *Appl. Anim. Behav. Sci.* 109(2-4):355-373.

5. Lansade L, Pichard G, Leconte M (2008) Sensory sensitivities: Components of a horse's temperament dimension. *Appl. Anim. Behav. Sci.* 114(3-4):534-553.

6. Wade CM*, et al.* (2009) Genome Sequence, Comparative Analysis, and Population Genetics of the Domestic Horse. *Science* 326(5954):865-867.

7. Moreews F, Rauffet G, Dehais P, Klopp C (2011) SigReannot-mart: a query environment for expression microarray probe re-annotations. *Database-the Journal of Biological Databases and Curation*.

8. Cole SW, Yan W, Galic Z, Arevalo J, Zack JA (2005) Expression-based monitoring of transcription factor activity: the TELiS database. *Bioinformatics* 21(6):803-810.

9. Sui SJH, Fulton DL, Arenillas DJ, Kwon AT, Wasserman WW (2007) oPOSSUM: integrated tools for analysis of regulatory motif over-representation. *Nucleic Acids Res.* 35:W245-W252.

10. Kwon AT, Arenillas DJ, Hunt RW, Wasserman WW (2012) oPOSSUM-3: Advanced Analysis of Regulatory Motif Over-Representation Across Genes or ChIP-Seq Datasets. *G3-Genes Genomes Genetics* 2(9):987-1002.
