# Supplementary material for: Behavioral and Transcriptomic Fingerprints of an Enriched Environment in Horses (Equus caballus)
Source: PLoS One. 2014 Dec 10;9(12):e114384. doi: 10.1371/journal.pone.0114384 (PMC4262392; doi:10.1371/journal.pone.0114384)
Supplement: Appendix S1 — Personality data. (PDF) [file pone.0114384.s006.pdf]

week5

group	animal number	***nb of contacts with passive human***	***latency to put halter***	***number of glances at***	***number of contact with***	***number of neighs during social***	***latensy to eat during novel***	***latency to eat during***	***number of sectors***	***response to von-Frey***	***reaction to stifle-haunch axis***	***handling test: nb of defensive***
EE treated	468	10	8	1	13	2	85	101	27	1	1,49	0
EE treated	469	9	8	5	11	8	75	55	50	1,5	1,5	0
EE treated	473	6	8	1	12	0	29	33	63	1	2,37	0
EE treated	475	9	8	4	0	0	180	180	45	2	1,36	0
EE treated	642	11	8	8	0	1	106	180	57	2,5	8	0
EE treated	643	15	8	2	15	4	51	29	56	2	2,67	0
EE treated	646	10	8	2	2	1	180	33	44	2,5	1,99	0
EE treated	648	15	8	1	7	7	24	5	72	3	4,12	0
EE treated	649	14	8	3	9	0	57	35	71	2,5	1,48	1
EE treated	651	19	8	2	25	1	180	180	63	3	2,87	1
Control	470	11	8	9	5	0	72	70	34	2	4	2
Control	471	0	30	11	0	3	56	140	63	3	1,99	4
Control	472	0	62	10	1	8	114	80	106	1	5,5	1
Control	474	0	80	4	2	0	180	141	39	2,5	9	2
Control	476	0	13	8	0	0	180	180	47	2,5	7,42	4
Control	477	2	15	9	4	0	7	180	135	3	12	4
Control	645	14	8	14	0	7	60	180	54	4	2,62	3
Control	647	9	37	1	25	2	16	180	70	2	5,87	5
Control	650	2	23	6	0	1	180	180	63	4	4,12	4

week12

group	animal number	***nb of contacts with passive human***	***latency to put halter***	***number of glances at novel object***	***number of contact with novel object***	***number of neighs during social isolation test***	***latensy to eat during novel area test***	***latency to eat during suddenness test***	***number of sectors crossed***	***response to von-Frey filaments***	***reaction to stifle-haunch axis stimulation***
EE treated	468	11	8	2	20	1	11	180	26	0	1,37
EE treated	469	1	8	7	0	3	11	180	35	1	1,79
EE treated	473	1	8	4	7	1	9	8	94	1	3,04
EE treated	475	8	8	2	8	0	180	108	41	0,5	1,5
EE treated	642	14	8	4	9	0	52	12	41	1,5	4,12
EE treated	643	8	8	6	11	2	18	13	65	1,5	2,25
EE treated	646	12	8	4	6	0	20	180	56	1	3,5
EE treated	648	16	8	1	12	3	8	3	66	1	2,37
EE treated	649	9	8	5	8	2	14	0	47	2	0,5
EE treated	651	13	8	3	22	1	15	21	72	3,5	1,75
Control	470	4	8	15	6	0	180	180	21	1	2,92
Control	471	4	14	13	0	1	29	180	55	4	5,25
Control	472	5	12	13	0	7	180	180	54	2	5,74
Control	474	4	60	5	11	0	180	7	26	2,5	10,5
Control	476	0	18	8	4	0	180	8	49	2	5,25
Control	477	0	10	10	0	0	180	180	64	2	10
Control	645	2	8	5	5	3	180	170	44	3,5	2
Control	647	16	8	10	10	0	28	180	33	3	3,75
Control	650	0	14	11	0	0	180	180	39	2,5	4,5

week 23	animal number	***nb of contacts with passive human***	***latency to put halter***	***number of glances at novel object***	***number of contact with novel object***	***number of neighs during social isolation test***	***latensy to eat during novel area test***	***latency to eat during suddenness test***	***number of sectors crossed***	***response to von-Frey filaments***	***reaction to stifle-haunch axis stimulation***
EE treated	468	5	8	0	10	1	11	74	52	0	0,75
EE treated	469	0	8	1	8	6	180	180	40	0	0,75
EE treated	473	6	8	2	7	1	19	37	64	1	1,25
EE treated	475	4	8	3	0	0	180	180	47	1	1,125
EE treated	642	10	8	4	15	4	26	7	83	2	4,425
EE treated	643	0	8	4	17	5	21	180	53	3	1,25
EE treated	646	16	8	1	9	3	90	165	74	2	2,125
EE treated	648	7	8	0	13	6	9	5	70	1,5	1,75
EE treated	649	15	8	1	19	6	20	180	86	2,5	1,5
EE treated	651	10	8	0	33	3	33	180	27	3,5	1,5
Control	470	13	8	9	2	1	36	180	33	0,5	2,67
Control	471	9	8	9	0	2	34	180	81	3	2,5
Control	472	11	8	4	11	7	95	180	130	2	3,175
Control	474	13	8	8	9	6	180	16	59	1	5,425
Control	476	0	19	7	0	1	180	180	32	2	3,675
Control	477	5	8	8	3	1	180	180	107	2,5	7,5
Control	645	7	8	7	3	3	180	11	45	3	2,5
Control	647	12	8	2	21	0	39	180	85	2	1,25
Control	650	13	8	14	0	1	180	180	84	2,5	2,675
