# Supplementary material for: Behavioral and Transcriptomic Fingerprints of an Enriched Environment in Horses (Equus caballus)
Source: PLoS One. 2014 Dec 10;9(12):e114384. doi: 10.1371/journal.pone.0114384 (PMC4262392; doi:10.1371/journal.pone.0114384)
Supplement: Appendix S2 — Learning data. (PDF) [file pone.0114384.s007.pdf]

appendix S2: percentage of success during the learning tests

		percentage of success during the learning tests			
		shaping phase		Go/no-Go task	
animal number	group	A+	B+	A+	B-
468	EE treated	75,00	75,00	41,84	36,08
469	EE treated	100,00	66,67	28,57	24,36
473	EE treated	50,00	100,00	98,33	68,85
475	EE treated	75,00	100,00	80,23	64,77
642	EE treated	60,00	75,00	95,88	90,72
643	EE treated	71,43	57,14	92,42	79,41
648	EE treated	87,50	50,00	49,32	54,79
649	EE treated	75,00	75,00	87,88	83,33
651	EE treated	75,00	75,00	100,00	50,00
470	Control	100,00	100,00	86,49	62,16
471	Control	80,00	50,00	89,00	76,24
472	Control	50,00	75,00	94,52	79,45
474	Control	60,00	100,00	65,00	73,77
476	Control	66,67	100,00	87,63	84,54
477	Control	66,67	66,67	92,31	84,85
645	Control	52,63	31,58	47,50	56,41
647	Control	100,00	100,00	100,00	40,00
650	Control	75,00	75,00	96,64	73,33
