# Supplementary material for: Behavioral and Transcriptomic Fingerprints of an Enriched Environment in Horses (Equus caballus)
Source: PLoS One. 2014 Dec 10;9(12):e114384. doi: 10.1371/journal.pone.0114384 (PMC4262392; doi:10.1371/journal.pone.0114384)
Supplement: Appendix S3 — Cortisol data. (PDF) [file pone.0114384.s008.pdf]

group	horse n°	week	morning/afternoon	salivary cortisol (ng/ml)
Control	470	0	morning	
Control	471	0	morning	0,85
Control	472	0	morning	0,77
Control	474	0	morning	0,79
Control	476	0	morning	0,91
Control	477	0	morning	0,78
Control	645	0	morning	1,10
Control	647	0	morning	0,54
Control	650	0	morning	0,82
EE-treated	468	0	morning	1,12
EE-treated	469	0	morning	0,59
EE-treated	473	0	morning	0,71
EE-treated	475	0	morning	0,73
EE-treated	642	0	morning	
EE-treated	643	0	morning	1,11
EE-treated	646	0	morning	0,62
EE-treated	648	0	morning	0,93
EE-treated	649	0	morning	0,63
EE-treated	651	0	morning	0,54
Control	470	0	afternoon	
Control	471	0	afternoon	0,45
Control	472	0	afternoon	0,47
Control	474	0	afternoon	0,39
Control	476	0	afternoon	0,71
Control	477	0	afternoon	0,30
Control	645	0	afternoon	0,89
Control	647	0	afternoon	0,35
Control	650	0	afternoon	0,59
EE-treated	468	0	afternoon	0,73
EE-treated	469	0	afternoon	0,30
EE-treated	473	0	afternoon	0,30
EE-treated	475	0	afternoon	0,56
EE-treated	642	0	afternoon	
EE-treated	643	0	afternoon	0,61
EE-treated	646	0	afternoon	0,49
EE-treated	648	0	afternoon	0,63
EE-treated	649	0	afternoon	0,41
EE-treated	651	0	afternoon	0,74
Control	470	6	morning	0,43
Control	471	6	morning	0,39
Control	472	6	morning	0,44
Control	474	6	morning	0,30
Control	476	6	morning	0,66
Control	477	6	morning	0,30
Control	645	6	morning	0,30
Control	647	6	morning	0,30
Control	650	6	morning	0,54
EE-treated	468	6	morning	0,49

EE-treated	469	6	morning	0,81
EE-treated	473	6	morning	1,03
EE-treated	475	6	morning	0,89
EE-treated	642	6	morning	0,52
EE-treated	643	6	morning	0,64
EE-treated	646	6	morning	0,89
EE-treated	648	6	morning	1,15
EE-treated	649	6	morning	1,08
EE-treated	651	6	morning	1,10
Control	470	6	afternoon	0,30
Control	471	6	afternoon	0,57
Control	472	6	afternoon	1,09
Control	474	6	afternoon	0,63
Control	476	6	afternoon	0,41
Control	477	6	afternoon	0,83
Control	645	6	afternoon	1,01
Control	647	6	afternoon	1,39
Control	650	6	afternoon	0,47
EE-treated	468	6	afternoon	0,59
EE-treated	469	6	afternoon	0,65
EE-treated	473	6	afternoon	0,88
EE-treated	475	6	afternoon	0,65
EE-treated	642	6	afternoon	0,50
EE-treated	643	6	afternoon	1,06
EE-treated	646	6	afternoon	0,50
EE-treated	648	6	afternoon	1,14
EE-treated	649	6	afternoon	1,21
EE-treated	651	6	afternoon	0,60
Control	470	12	morning	0,48
Control	471	12	morning	0,57
Control	472	12	morning	0,39
Control	474	12	morning	0,5
Control	476	12	morning	0,76
Control	477	12	morning	
Control	645	12	morning	0,77
Control	647	12	morning	0,57
Control	650	12	morning	0,83
EE-treated	468	12	morning	0,63
EE-treated	469	12	morning	0,76
EE-treated	473	12	morning	1,34
EE-treated	475	12	morning	0,51
EE-treated	642	12	morning	0,64
EE-treated	643	12	morning	0,59
EE-treated	646	12	morning	0,42
EE-treated	648	12	morning	0,56
EE-treated	649	12	morning	1,92
EE-treated	651	12	morning	0,91
Control	470	12	afternoon	0,31
Control	471	12	afternoon	0,39
Control	472	12	afternoon	0,69

Control	474	12	afternoon	0,3
Control	476	12	afternoon	0,37
Control	477	12	afternoon	0,30
Control	645	12	afternoon	0,62
Control	647	12	afternoon	0,33
Control	650	12	afternoon	0,63
EE-treated	468	12	afternoon	1,31
EE-treated	469	12	afternoon	0,54
EE-treated	473	12	afternoon	2,07
EE-treated	475	12	afternoon	0,78
EE-treated	642	12	afternoon	0,31
EE-treated	643	12	afternoon	0,86
EE-treated	646	12	afternoon	0,31
EE-treated	648	12	afternoon	0,29
EE-treated	649	12	afternoon	0,6
EE-treated	651	12	afternoon	0,32
